# SORT1 promote the metastasis and invasion of hepatocellular carcinoma via p38/β-catenin/ZEB1 signaling pathway

**DOI:** 10.1038/s41419-025-07871-y

**Published:** 2025-08-01

**Authors:** Hongjie Chen, Wei Liao, Yuanhui Jiang, Guichan Liao, Xinrui Gao, Siyi Cen, Lili Liu, Jie Peng, Cai Shaohang

**Affiliations:** 1 State Key Laboratory of Organ Failure Research, Guangdong Provincial Key Laboratory of Viral Hepatitis Research, Key Laboratory of Infectious Diseases Research in South China, Department of Infectious Diseases, Nanfang Hospital, Southern Medical University, Guangzhou, China; 2 Department of Intensive Care Unit, State Key Laboratory of Oncology in South China, Collaborative Innovation Center for Cancer Medicine, Sun Yat-sen University Cancer Center, Guangzhou, China; 3 Department of Infectious Disease, Center of Scientific Research, Maoming People’s Hospital, Maoming, China; 4 Southern Medical University, Guangzhou, China; 5 Department of Pathology, State Key Laboratory of Oncology in South China, Sun Yat-sen University Cancer Center, Guangzhou, China

**Keywords:** Metastasis, Oncogenes

## Abstract

Metastasis is the predominant reason for high mortality of hepatocellular carcinoma (HCC) patients. Understanding the molecular mechanisms underlying HCC metastases is critical. Here, we reported that SORT1 functioned as an oncogene by facilitating HCC metastasis. Elevated SORT1 expression was positively correlated with increased tumor number, advanced TNM stage, and vascular invasion. Mechanistically, SORT1 binds to p38 and enhances its stability. Furthermore, p38 phosphorylates GSK-3β at Ser9, which promotes nuclear accumulation of β-catenin, leading to the transcription of ZEB1 and secretion of exosomes. Knockdown of SORT1 and ZEB1 inhibited HCC metastasis, whereas upregulation of ZEB1 restored SORT1 knockdown-induced suppression of HCC metastasis. SORT1 expression was positively correlated with Sterol regulatory element-binding proteins 2 (SREBP2) and ZEB1 expression in human HCC tissues. In light of these results, SORT1 as a potential prognostic biomarker in HCC and targeting WNT/β-catenin signaling pathway, could be an effective therapeutic strategy against HCC metastasis.

## Introduction

According to the latest global cancer statistics, hepatocellular carcinoma (HCC) ranked as the sixth most commonly diagnosed cancer and the fourth leading cause of cancer death worldwide in 2018 [1]. Since HCC is often diagnosed at an advanced stage, the long-term prognosis for HCC remains extremely poor, with 5-year overall survival rates of 21–22% [2]. However, survival rates can reach 70% with surgical resection or transplantation in early-stage patients [3], which indicates that metastasis being the major reason for the poor survival of HCC patients. Thus, identification of critical molecules that contribute to the metastasis and invasion phenotype of HCC, and clarification of the underlying molecular mechanism are urgently needed to improve HCC prognosis.

SORT1, also known as neurotensin receptor-3, is a member of the vacuolar protein sorting 10 (VPS10) protein family of sorting receptors [4]. SORT1 plays important roles in intracellular trafficking and sorting for various ligands, including neurotrophic factors and neuropeptides, cytokine receptors, tyrosine receptor kinases, G-protein coupled receptors, and ion-channels [5–8] between the plasma membrane and the trans-Golgi network (TGN). Deregulation of SORT1 function has been implicated in the development and progression of neurological and cardiovascular disease [9–11]. Recently, SORT1 has also been found in different types of human cancer cells and tissues, including breast [12], pancreatic cancer [13], lymphocytic leukemia [14], and glioblastoma [15, 16], which suggests a role for this protein in tumorigenesis and progression. Ahn HR et al. revealed SORT1’s role in promoting HCC metastasis via Notch/CD133 signaling [17], establishing its prognostic value. However, the precise regulatory mechanisms underlying SORT1-driven hepatocarcinogenesis and its crosstalk with other oncogenic pathways require deeper exploration.

Accordingly, we performed a retrospective clinical investigation of SORT1 expression, and the expression of SORT1 is significantly upregulated in HCC primary tumors compared with para-carcinoma tissue, which is correlated with poor prognosis of HCC patients. Further, we conducted in vivo and in vitro experiments to assess whether SORT1 is an oncogene for HCC. We observed that SORT1 upregulation increases tumor cell metastasis and invasion by P38/β-catenin/ZEB1 axis.

## Results

### SORT1 expression is increased and associated with poor prognosis in hepatocellular carcinoma

**Fig. 1:**
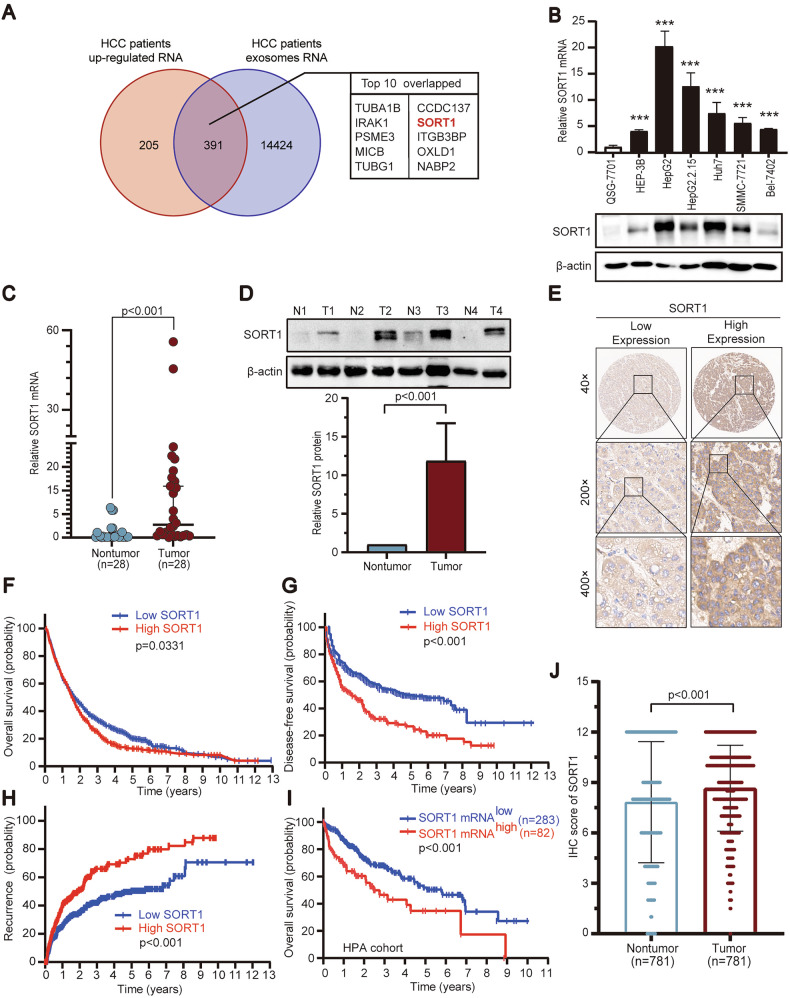
SORT1 is up-regulated in hepatocellular carcinoma and associated with disease progression. **A** Venn diagram showing overlapped genes between exosomal RNA of HCC patients in the EV databases Exocarta and up-regulated RNA of HCC patients. **B** mRNA and protein expression of SORT1 in HCC and immortalized liver cell lines were examined by a quantitative RT-PCR and western blotting. **C** Comparative analysis of SORT1 mRNA expression in 28 paired HCC and corresponding adjacent liver tissue samples. **D** Western blot detection of SORT1 protein expression in four paired HCC and adjacent nontumor tissues. Relative protein levels were quantified as fold-changes compared to nontumor tissues. **E** Expression of SORT1 was determined by a TMA-based immunohistochemistry. Representative images of tumor with low and high SORT1 expression are shown. **J** The IHC scores were shown and were analyzed statistically. Correlation of SORT1 expression and overall survival (**F**), disease-free survival (**G**) and recurrence (**H**) was determined in a cohort of 781 patients by Kaplan–Meier analysis. **I** The probability of overall survival in HCC patients expressing high or low SORT1 levels was assessed using the Human Protein Atlas database.

Integrative analysis of long-read sequencing data from 8 paired HCC tumor/adjacent tissues and exosomal RNA profiles from EV databases Exocarta identified SORT1 as a top 10 overlapping gene among tumor-upregulated transcripts (Fig. 1A). Then, we determined SORT1 expression in HCC cell lines and fresh tissue samples by quantitative RT-PCR and western blotting. The results showed that SORT1 mRNA and protein levels in most of HCC cell lines were significantly higher than those in immortalized hepatic cell lines QSG-7701 (Fig. 1B). In 28 pairs of HCC fresh tissues, SORT1 mRNA was increase compared to matched nontumor tissues (Fig. 1C). Consistent with this, the SORT1 protein level was significantly increase by 11.8-fold in 4 HCC specimens (Fig. 1D). To determine the clinical significance of SORT1 in HCC, we next examined the expression of SORT1 in a TMA cohort consisting of 781 HCC patients. SORT1 was primarily located in the cytoplasm and significantly up-regulated in HCC tissues (Fig. 1E, J). Statistical analysis showed that higher SORT1 expression was associated with tumor multiplicity, higher TNM stage and the presence of vascular invasion (Table S3). Kaplan–Meier analysis showed that HCC patients with higher SORT1 expression had a shorter overall, disease-free survival and higher recurrence rate (Fig. 1F, G, H). The Human Protein Atlas (HPA) database showed a cohort of 365 HCC patients and patients with high expression of SORT1 experienced a shorter period of overall survival (Fig. 1I). Multivariate analyses indicated SORT1 as an independent prognostic factor of disease-free survival and tumor recurrence in HCC (Tables S5 and S6). Taken together, our data suggest that SORT1 may serve as a promising biomarker for prognosis in HCC.

### SORT1 correlated with metastatic features towards hepatocellular carcinoma cells

**Fig. 2:**
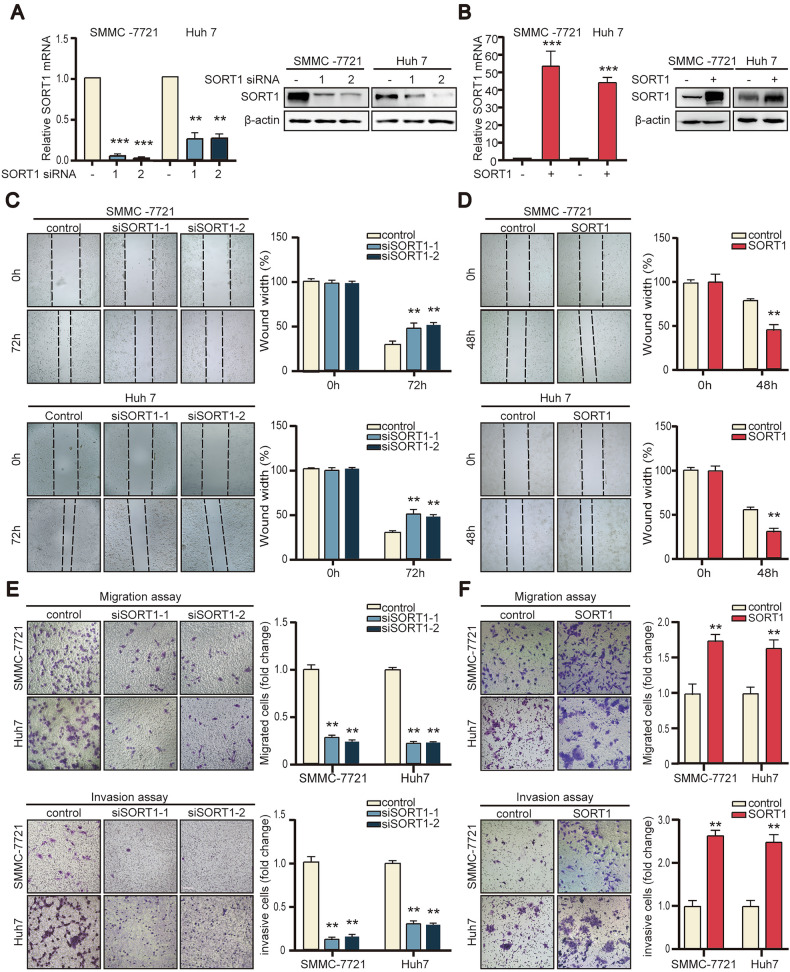
SORT1 promotes hepatocellular carcinoma cell migration and invasion in vitro. **A** SORT1 was silenced in SMMC-7721 and Huh-7 cells by siRNAs. **B** SORT1 was overexpressed by transfecting pcDNA3.1-SORT1 plasmids in SMMC-7721 and Huh-7 cells. Transwell assays detected the invasion and migration abilities in cells with SORT1 depletion **C** or overexpression (**D**). Representative images and the quantitative data of three randomly selected fields are shown. Wound-healing assays measuring cell motility in SORT1-knockdown (**E**) and SORT1-expressing cells (**F**). Scale bar = 200 μm. Quantitative data are presented as the mean ± SD. *p < 0.05; **p < 0.01; ***p < 0.001.

Next, given the need for bidirectional SORT1 modulation, we performed in vitro functional assays using SMMC-7721 and Huh7 cell lines (moderate expression range) to explore its biological role in HCC progression. SORT1 was either knocked down or overexpressed in SMMC-7721 and Huh-7 cells (Fig. 2A, B). In light of the clinicopathological data suggesting SORT1 participates in HCC progression, we prioritized the investigation of its role in tumor dissemination. Wound-healing assays further demonstrated that SORT1-overexpressing cells filled up the wound faster than control cells (Fig. 2D). By contrast, down-regulation of SORT1 expression hindered cell movement (Fig. 2C). Transwell assays also showed that the down-regulation of SORT1 reduced, whereas the exogenous expression of SORT1 enhanced the ability of cell invasion and migration in HCC cells (Fig. 2E, F). Meanwhile, we assessed proliferation under knockdown conditions using MTT assays (Supplementary Fig. 1A), EdU incorporation assays (Supplementary Fig. 1B) and PI staining (Supplementary Fig. 1C). No significant changes in proliferation were detected, suggesting SORT1 depletion does not directly regulate HCC cell growth.

### SORT1 triggers WNT/β-catenin pathway via p38

**Fig. 3:**
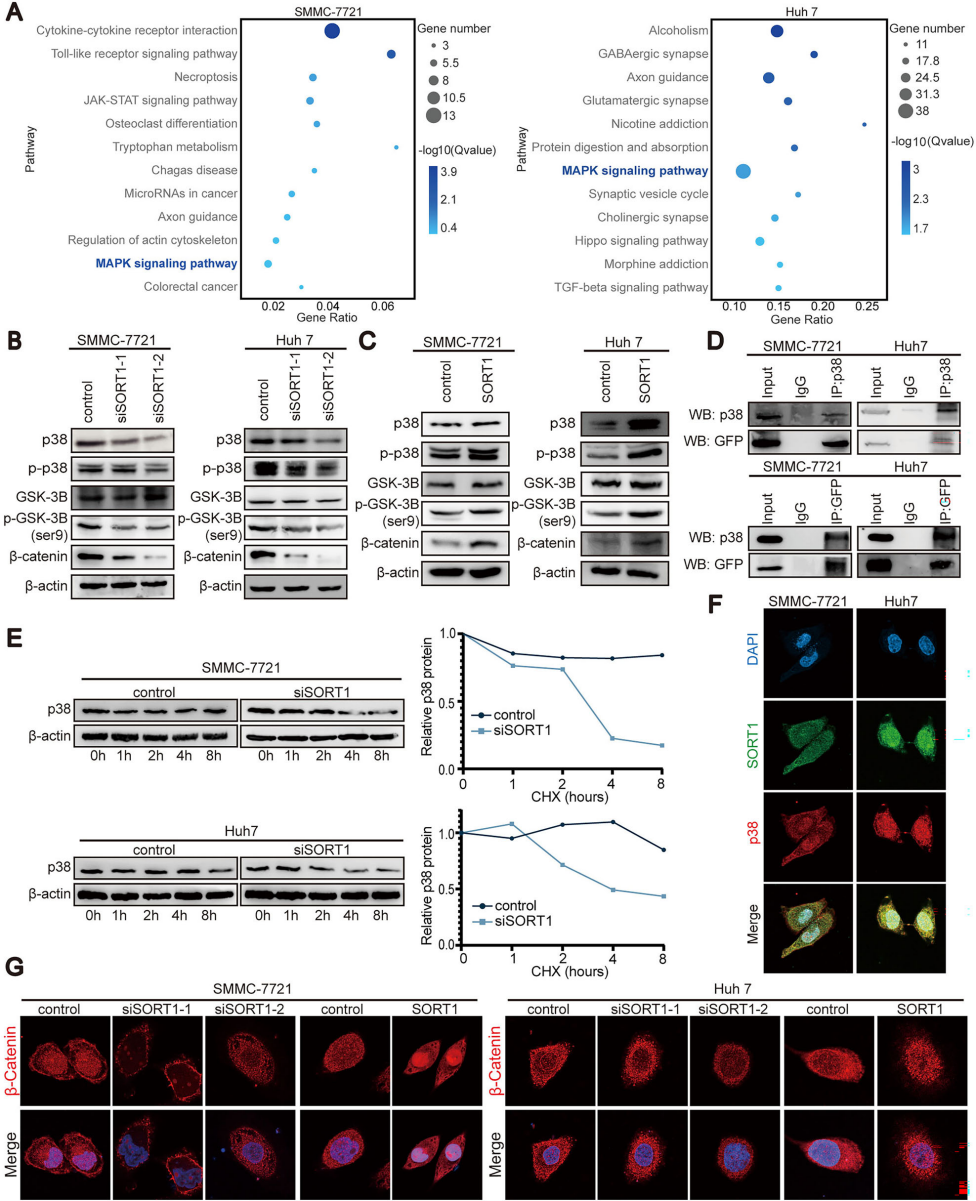
SORT1 triggers the WNT/β-catenin pathway via binding with p38 in hepatocellular carcinoma cells. **A** Bubble map of KEGG pathway analyses in SMMC-7721 and Huh7 based on SORT1 knock down transcriptome profiling. **B**, **C** Proteins obtained from HCC cells up-regulated or knock down SORT1 were subjected to Western blot analysis to examine the activation of the WNT/β-catenin pathway. **D** Co-immunoprecipitation (Co-IP) assays demonstrating direct interaction between SORT1 and p38 in both HCC cell lines. **E** SORT1-depleted cells were treated with 50 μg/ml CHX for the indicated time period, and p38 stability was determined by western blotting. Protein degradation rates were calculated from densitometric analysis of western blot bands. **F** Immunofluorescence staining was performed to indicate the co-localization between SORT1 (green) and p38 (red). DAPI was used to stain the nucleus. **G** Immunofluorescence staining was performed to indicate the cellular localization of β-catenin. DAPI was used to stain the nucleus.

To identify potential underlying mechanisms regulated by SORT1 in HCC, we performed RNA sequencing transcriptional profiling on SORT1-knockdown SMMC-7721/Huh-7 cells. Pathway enrichment analyses marked suppression of MAPK signaling pathways upon SORT1 depletion (Fig. 3A). We then performed systematic validation of all three MAPK subpathways (ERK, JNK, p38) in HCC cells through western blot analysis. Knockdown of SORT1 decreased p38, phospho-p38, GSK-3β, phospho-GSK-3β (Ser 9) and β-catenin expression in SMMC-7721 and Huh-7 cells (Fig. 3B), while ERK/JNK markers remained unaffected (Supplementary Fig. 3A). Notably, no significant changes in total AKT or phospho-AKT levels following SORT1 knockdown in both SMMC-7721 and Huh7 cells (Supplementary Fig. 3A). We also confirmed that ectopic expression of SORT1 increased the expression of these mentioned proteins levels (Fig. 3C). We treated HCC cells with protein synthesis inhibitor (cycloheximide) and found that down-regulating SORT1 significantly decreased the half-life of endogenous p38 (Fig. 3E). Moreover, our co-immunoprecipitation (IP) results confirmed that the endogenous p38 binds with SORT1 in SMMC-7721 and Huh-7 cells (Fig. 3D). We further determined the colocalization of SORT1 and p38 in the cytoplasm of HCC cells by confocal immunofluorescence (Fig. 3F). Additionally, we performed rigid protein–protein docking to predict binding modes between SORT1 and p38 (ZDOCK scores = 1235.845) (Supplementary Fig. 3E). Interestingly, SORT1 overexpression significantly promoted β-catenin nuclear translocation, as demonstrated by immunofluorescence staining (Fig. 3G). Furthermore, nuclear localization of β-catenin can be restored by p38 overexpression in SORT1-knockdown HCC cells (Supplementary Fig. 3C).

### SORT1 augments hepatocellular carcinoma metastasis and invasion potential via WNT/β-catenin pathway

**Fig. 4:**
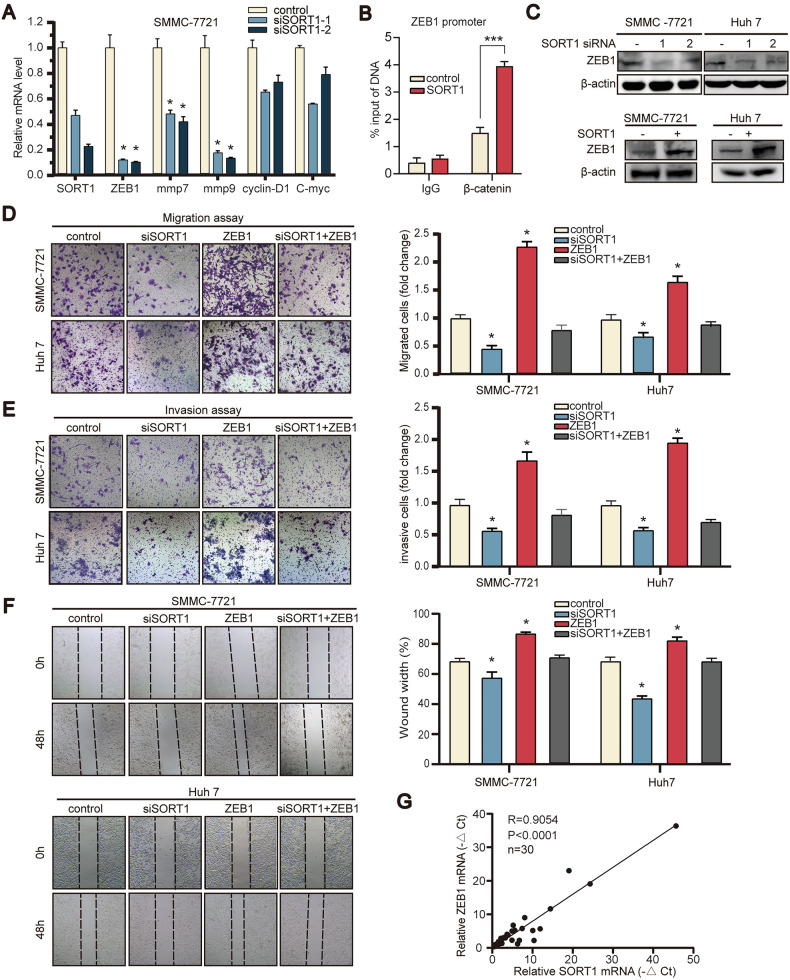
SORT1 promote metastasis by up-regulate ZEB1 in vitro. **A** RNA expression in SORT1-konckdown SMMC-7721 cell. **B** Chromatin immunoprecipitation (ChIP) assays demonstrating β-catenin recruitment to the ZEB1 promoter in SORT1-overexpressing cells. Enrichment fold changes were normalized to IgG controls. **C** The protein expression of ZEB1 in SMMC-7721 and Huh-7 cells with SORT1 suppression and overexpression by western blotting. Rescue experiments via ZEB1 overexpression in SORT1-knockdown cells: Matrigel-based Transwell assays evaluating invasion (**D**) and migration (**E**) capacities under ZEB1 rescue conditions. **F** Wound-healing assays demonstrated cell movement capacity in siSORT1 or the siNC along SMMC-7721 and Huh-7 cells treated with the ZEB1-plasmid or negative control. **G** The relationship between SORT1 mRNA and ZEB1 mRNA was determined in 30 HCC clinical specimens. R Pearson correlation coefficient. Quantitative data are presented as the mean ± SD. *p < 0.05; **p < 0.01; ***p < 0.001.

We then examined the downstream changes in gene expression associated with WNT/β-catenin pathway in SORT1 down-regulated HCC cells (Fig. 4A). ZEB1 was selected as a candidate target of SORT1 for further investigation. To determine whether β-catenin directly regulates ZEB1 transcription upon SORT1 modulation, we performed chromatin immunoprecipitation (ChIP) assays using β-catenin-specific antibodies in SORT1-overexpressing SMMC-7721 cells. Quantitative PCR analysis revealed significant enrichment of β-catenin binding to the ZEB1 promoter region in SORT1-overexpressing cells compared to controls (fold enrichment: 2.62, *p* < 0.001; Fig. 4B). ZEB1 protein expression in SMMC-7721 and Huh-7 cells was markedly decreased and increased with SORT1 silencing and overexpression, respectively (Fig. 4C). To determine whether the effects of SORT1 on cell migration and invasion were ZEB1-dependent, wound-healing assays and transwell assays were performed. Transwell assays showed that down-regulated SORT1-mediated migration and invasion was recovered by ZEB1 overexpressed (Fig. 4D, E). We also found that ZEB1 overexpression partially restored the wound healing capability of SMMC-7721 and Huh-7 cells, and led to reversal of the facilitating effects of SORT1 knockdown on these processes (Fig. 4F). Additionally, we found that SORT1 RNA expression was positively correlated with the RNA expression of ZEB1 in HCC tissues (Fig. 4G). To further validate this finding, we have performed additional correlation analyses using two independent public databases (TIMER and GEPIA), which consistently confirm this association (Supplementary Fig. 3B, D).

### SORT1 promotes HCC metastasis in vivo

**Fig. 5:**
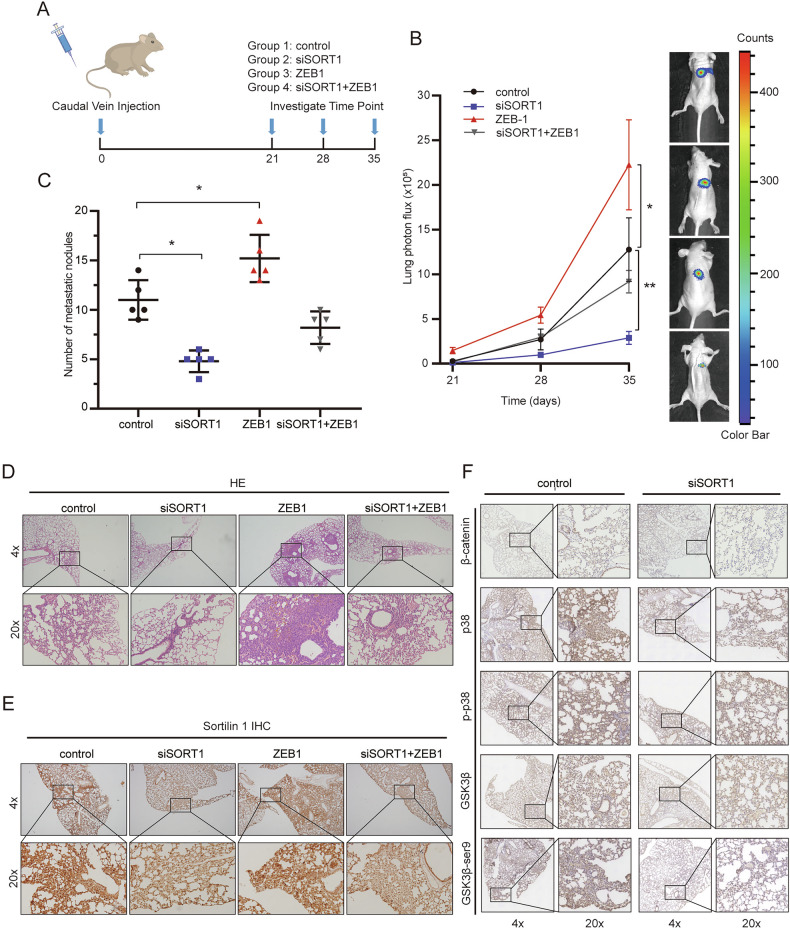
SORT1 promote metastasis by up-regulate ZEB1 in vivo. **A** SMMC 7721 cells labeled with luciferase were injected intravenously through the tail vein of nude mice to generate a commonly used model of lung metastatic lesions. **B** Luciferase signals of lung metastasis in SORT1-silencing and/or ZEB1 expressing group mice and control group mice; the data were expressed as mean ± SD. **C** Quantification of tumor metastatic nodules in lung tissues. **D** Representative H&E staining of sections with metastatic nodules lung tissues. **E** SORT1 immunohistochemistry of sections with metastatic nodules lung tissues. **F** Immunohistochemical profiles of β-catenin, GSK-3β, phospho-GSK-3β (Ser9), p38 phospho-p38 with metastatic nodules lung tissues. Quantitative data are presented as the mean ± SD. *p < 0.05; **p < 0.01; ***p < 0.001.

Caudal vein injection models were established to examine the effect of SORT1 on tumor metastasis in vivo (Fig. 5A). Imaging with bioluminescence was used to follow the growth of pulmonary metastases (Fig. 5B). Statistical analysis showed significantly lower luciferase signal in nude mice injected with luciferase-labeled SMMC-7721 cells stably down-expressing SORT1 than in mice injected with control. ZEB1 over-expression recovers the luciferase signal of SORT1-surpression HCC cells. Mice were sacrificed after 35 days after injection. The number of lung metastases was less frequent in the SORT1-deficient group and more frequently found in ZEB1 over-expression group. ZEB1 over-expression recovered tumors metastasized capability of SORT1-surpression (Fig. 5C). HE staining (Fig. 5D) and SORT1 immunohistochemistry (Fig. 5E) of the lung tissues also showed a similar result. To mechanistically link these phenotypic changes with pathway activity in vivo, we also perform IHC analysis for the pathway. SORT1 knockdown reduced expression of β-catenin and decreased phosphorylation levels of GSK-3β (Ser9) and p38 in metastatic lesions (Fig. 5F), which is consistent with our in vitro results.

### SORT1 increase metastasis and invasion by triggers EMT and exosomal MMP9 protein

**Fig. 6:**
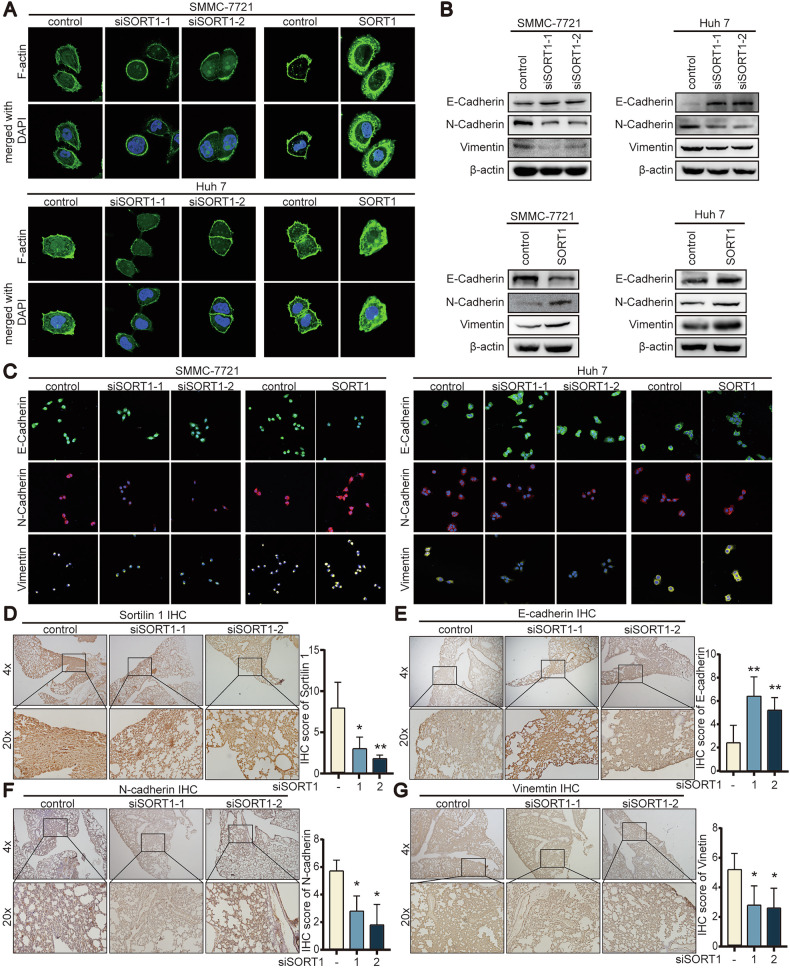
SORT1 promotes the EMT process. **A** SMMC-7721 and Huh-7 cells were introduced with SORT1 knock-down or overexpression vector and F-actin (green) was used to indicate the cell morphology. Scale bar = 20 μm. **B** Expression of EMT-related markers, E-cadherin, vimentin, and N-cadherin was determined by western blotting in cells with SORT1 silencing or overexpression. **C** Expression of EMT-related markers, E-cadherin, N-cadherin, and vimentin were detected by immunofluorescence staining in cells with SORT1 silencing or overexpression. **D**–**G** SORT1 and EMT-related markers, E-cadherin, N-cadherin, and vimentin were detected in immunohistochemistry metastatic nodules in lung tissues. Quantitative data are presented as the mean ± SD. *p < 0.05; **p < 0.01; ***p < 0.001.

To clarify the mechanism of SORT1 was involved in HCC metastasis, cell shape was identified by using phallotoxin staining to visualize cytoskeleton F-actin. The formation of pseudopodium, which improved motility of tumor cell, was increased in SORT1-expressing cells but decreased in SORT1-depleted cells (Fig. 6A). Further immunofluorescence staining and western blotting showed that loss of SORT1 suppressed the EMT process by increasing the expression of epithelial marker E-cadherin and reducing the expression of mesenchymal markers N-cadherin and vimentin (Fig. 6B). Consistent with this, SORT1 overexpression down-regulated E-cadherin and up-regulated N-cadherin and vimentin (Fig. 6C). Immunohistochemistry of mice lungs demonstrated similar results (Fig. 6D, E, F, G). Finally, we isolated exosomes from the HCC cell supernatant and observed under a scanning electron microscope (Supplementary Fig. 2C). Exosome size distribution analyses revealed that exosome secreting by SORT1-depleted HCC cells decreased in particle diameter (Supplementary Fig. 2A, B). The efficiency of exosome purification were shown in Supplementary Fig. 2D. Additionally, we found a drop of exosomal MMP9 protein secreting from SORT1 down-regulated HCC cells (Supplementary Fig. 2E). To further validate the functional impact of SORT1-modified exosomes on tumor metastasis, we conducted exosome transfer experiments. Exosomes isolated from SORT1-overexpressing SMMC-7721 and Huh-7 cells were introduced into recipient HCC cells cultured. Remarkably, wound-healing assays demonstrated that treatment with SORT1-enriched exosomes significantly enhanced migratory capacity in both SMMC-7721 and Huh-7 cell lines compared to control exosomes (Supplementary Fig. 2F). Considering the function of SORT1 is intracellular trafficking between the plasma membrane and TGN, we then measured the Golgi by immunofluorescence. Golgi was smaller in SORT1 down-regulated HCC cells compared with control (Supplementary Fig. 2G). To further evaluate the clinical relevance of exosomal MMP9, we systematically analyzed clinical data from the ExoRBase exosome database. Notably, exosomal MMP9 levels were significantly elevated (*p* < 0.001) in HCC patients compared to healthy controls (Supplementary Fig. 2H).

### SREBP2 is an upstream regulator of SORT1 and associated with poor outcome

**Fig. 7:**
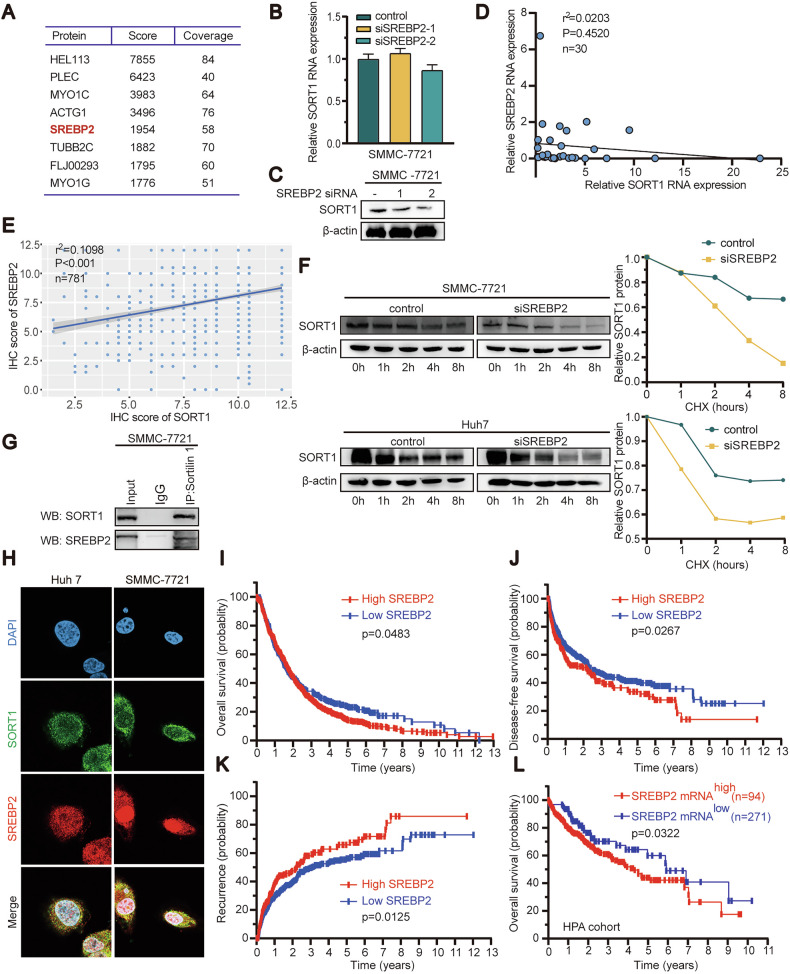
SREBP2 binding with SORT1 and has an impact on HCC prognostics. **A** Proteomic profiling identifies SREBP2 as a top candidate interacting partner of SORT1. Expression of SORT1 were determined by qPCR (**B**) and western blotting (**C**) in SREBP2 silencing SMMC 7721 cells. **D** Correlation between SREBP2 and SORT1 RNA expression in HCC tissues (*n* = 30). **E** Positive correlation between SREBP2 and SORT1 IHC score in HCC tissues (*n* = 781). **F** SREBP2-depleted cells were treated with 50 μg/ml CHX for the indicated time period, and SORT1 stability was determined by WB. **G** Co-IP of SREBP2 and SORT1 in cell lysates were from SMMC-7721 cells. **H** Subcellular co-localization was performed to indicate the co-localization between SREBP2 (red) and SORT1 (green). Correlation of SREBP2 expression and overall survival (**I**), disease-free survival (**J**), and recurrence (**K**) were determined in a cohort of 781 patients by Kaplan–Meier analysis. **L** The probability of overall survival in HCC patients expressing high or low SREBP2 levels was assessed using the Human Protein Atlas database.

Immunoprecipitation mass spectrometry (IP-MS) was performed in SMMC-7721 cells with endogenous proteins and showed SREBP2 binds SORT1 (Fig. 7A). We found that knockdown of SREBP2 in SMMC-7721 cells does not impact SORT1 in mRNA levels (Fig. 7B) but downregulated SORT1 protein (Fig. 7C). Similar results were found in RNA expression and IHC score of HCC patients (Fig. 7D, E). HCC cells treated with cycloheximide showed a significant decrease in SORT1 half-life when SREBP2 was repressed (Fig. 7F). The computational program of protein-protein docking was employed between SREBP2 and SORT1 (ZDOCK scores = 1314.695) (Supplementary Fig. 3G). Further, co-IP results and confocal immunofluorescence confirmed that the endogenous SREBP2 binds with SORT1 (Fig. 7G, H). To reinforce this observation, cross-validation analyses through two independent public platforms (TIMER and GEPIA) revealed a robust correlation between SREBP2 and SORT1 expression in HCC cohorts (Supplementary Fig. 3F, H). Kaplan–Meier analysis showed that HCC patients with higher SREBP2 expression had a lower overall, disease-free survival and higher recurrence rate (Fig. 7I, J, K). In the HPA’s cohort of 365 HCC patients, patients with high expression of SREBP2 experienced a shorter period of overall survival (Fig. 7L).

## Discussion

**Fig. 8:**
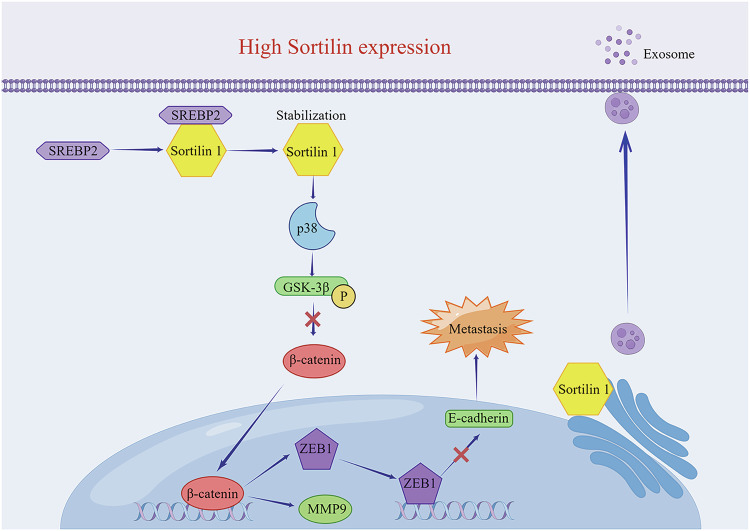
Schematic regulatory network of the SREBP2/SORT1/p38/β-catenin/ZEB1 signaling axis responsible for HCC metastasis. SORT1 is stabilized by SREBP2 and subsequently binds to p38. The accumulated p38 phosphorylates GSK3β at Ser9, which activates the β-catenin pathway. This activation transcriptionally upregulates ZEB1 expression and promotes exosomal MMP9 protein secretion, ultimately facilitating HCC metastasis. This Figure was created by Figdraw (www.figdraw.com).

Metastasis is an important hallmark of cancer and contributes the majority of HCC-associated deaths. Although several biomarkers have been identified, the underlying cellular and molecular mechanisms driving HCC metastasis progression are not known. In the present study, we demonstrate that high SORT1 expression was significantly associated with malignant features and exerted pro-metastatic activity towards HCC metastasis. The data represent a novel SREBP2/SORT1/p38/β-catenin/ZEB1 signaling axis promotes HCC metastasis via the EMT process and exosomal MMP9 protein (Fig. 8).

SORT1 was linked to oncogenesis and associated with poor prognosis, has been reported in most human cancers. We provide compelling biological and clinical evidence that SORT1 is overexpressed in HCC. A study on gliomas also showed SORT1 expression levels were significantly elevated in high-grade gliomas, and the expression levels were positively correlated with tumor malignancy [18]. In another study, a significant expression of SORT1 on the surface of PBMCs in chronic lymphocytic leukemia patients [14]. However, SORT1 was considered as an anti-oncogene in lung cancer by inhibiting the EGFR signaling pathway [19, 20]. Our data showed that SORT1 was frequently associated with increased tumor number, advanced TNM stage, and vascular invasion. According to our and HPA’s cohorts, HCC patients with high SORT1 expression likely had shorter overall and disease-free survival than those with low SORT1. Collectively, these findings suggest that SORT1 misregulation is sensitive to human cancers and may represent a potential biomarker for clinical surveillance of tumor progression.

SORT1 can promote tumor metastasis by different mechanisms. A previous study showed migration features of human pancreatic ductal adenocarcinoma cell are mainly mediated through the SORT1/NTR3 receptor with modified adhesion abilities and activation of small Rho GTPases [21]. In HT29 colon cancer cells, SORT1 binding to the plasma membrane increased the intracellular calcium levels, which led to the activation of FAK–Src-dependent PI3 kinase pathway and weakened HT29 epithelial property to prove tumor metastasis [22, 23]. In the present study, we confirmed SORT1 promotes HCC cell migration and metastasis by activating the Wnt/β-catenin signal pathway. A previous study also reported that SORT1 promotes tumor metastasis via Wnt/β-catenin in glioblastoma [16]. Our data showed that ectopic SORT1 combined with p38, then induced GSK3β Ser9 phosphorylation and β-catenin protein stability, leading to the nuclear accumulation of β-catenin. According to research, p38 MAPK regulates canonical Wnt/β-catenin signaling by inactivation of GSK3β [24]. GSK3β is the key enzyme that suppresses Wnt/β-catenin signaling by direct coupling to the Wnt receptors with proteins APC and Axin [25], ultimately phosphorylating β-catenin and causing its degradation by the proteasome.

The dysregulation of Wnt/β-catenin signaling is responsible for HCC initiation and progression [26]. Overexpression of β-catenin has been documented in several studies, indicating that the protein is upregulated in more than half of HCC patients [27, 28]. In addition, WNT/β-catenin has been shown to be involved in HCC metastasis by triggering the expression of EMT activators, such as Twist, ZEB1, and Snail [16, 29, 30]. Our results demonstrated that SORT1 functionally promoted EMT through increasing the expression of ZEB1 in vivo and in vitro. β-catenin has been proved to be an upstream regulator of ZEB1 [31]. Taken together, these results suggest that SORT1 promotes EMT through the upregulation of ZEB1 expression.

SORT1 exerts its functions by facilitating intracellular trafficking and sorting between the Golgi and the cell membrane [32]. Notably, the trans-Golgi network coordinates with the endoplasmic reticulum (ER) to regulate early endosome formation and cargo sorting, which ultimately determines exosome composition and secretion efficiency [33]. A prior study demonstrated that SORT1 plays a critical role in exosome release in lung cancer cells, underscoring its conserved function in vesicular trafficking and exosome secretion [7]. According to our data, SORT1 enhances the synthesis and trafficking function of the Golgi, therefore promoting exosome and exosomal MMP9 protein secretion. It is known that matrix metalloproteinases (MMPs), including MMP7, MMP9, and MMP12, are secreted by both tumor and stromal cells, and they function to regulate metastasis by degrading extracellular matrix [34–37]. A previous study demonstrated that SORT1/LAMP2-mediated extracellular vesicle secretion and cell adhesion resulted in lenalidomide resistance in multiple myeloma [38]. Therefore, our data further confirmed the close relationship between SORT1-mediated exosome secretion and HCC cell invasiveness.

SREBP2 as an important transcription factor, belongs to the basic-helix-loop-helix leucine zipper class [39]. SREBP2 is known to target genes regulating glucose breakdown, cholesterol, and fatty acid synthesis, which may contribute to supporting the increased bioenergetic demand of proliferating and metastasis in tumor [40]. Our result also showed that SREBP2 correlates with poor outcomes in HCC. In the present study, we reveal that SREBP2 acts as an upstream regulator of SORT1 through binding in HCC cells. Further investigations are needed to elucidate the precise regulatory mechanism due to potential ER co-localization.

## Conclusion

In summary, we identified SORT1 as an oncogene with prognostic significance in HCC. SORT1 promotes HCC metastasis via a non-canonical mechanism, which triggers the p38/β-catenin pathway through transcriptionally activating the expression of ZEB1 and exosomal MMP9 protein secretion. Consequently, SORT1 might be a potential therapeutic target and prognostic biomarker for HCC patients.

## Materials and methods

### Patient samples

HCC tissue samples along with complete clinical and pathological data, were obtained from 781 patients diagnosed with HCC at Nanfang Hospital, Guangzhou, China. Informed consents were obtained from all patients. Information regarding the clinicopathological characteristics of HCC patients is presented in Table S3.

### RNA-seq analysis

The RNA libraries were sequenced on an Illumina sequencing platform by Genedenovo Biotechnology Co., Ltd. (Guangzhou, China). KEGG pathway enrichment analysis was performed using the OmicShare online tools platform (www.omicshare.com/tools).

### Bioinformatics

Exosomal RNA sequencing data from HCC patients were retrieved from the Exocarta database (http://www.exocarta.org). Exosomal MMP9 clinical data was analyzed from the ExoRBase exosome database (http://www.exorbase.org). Clinical survival data of HCC patients were obtained from the HPA database (https://www.proteinatlas.org). Two independent validation analyses were conducted using TIMER (http://timer.cistrome.org/) and GEPIA (http://gepia.cancer-pku.cn) databases to confirm the correlation patterns. All database queries were performed using default statistical parameters as specified in each platform’s analytical pipeline.

### Cell culture and reagents

Liver cancer cell lines HEP-3B, SMMC-7721, and Huh-7 were purchased from ATCC. QSG-7701 HepG2, HepG2.2.1,5, and Bel-7402 were obtained from the Cell Resource Center, Chinese Academy of Science Committee. All cell lines were grown in Dulbecco’s modified Eagle medium, supplemented with 10% fetal bovine serum. All the cell lines were incubated at 37 °C with 5% CO_2_. The cell line has recently been authenticated by sequencing identification method.

### Stable cell lines generation

SORT1 short hairpin RNA (shRNA) was constructed with the pLenti system and lentiviral plasmid overexpressing SORT1 was purchased from OBiO Technology (Shanghai) Corp. SMMC-7721 and Huh-7 cells were transfected either with pLenti or pLV plasmid, together with the third-generation lentiviral packaging system using Lipofectamine 3000 reagent (Thermo Fisher Scientific) according to the manufacturer’s instructions. At 48 h after infection, 2 μg/ml puromycin was added to obtain stable cell lines with successful transduction. The sequences of shRNAs used in this study are listed in Supplementary Table 1.

### Wound-healing assay and transwell assays

For wound-healing assay, plastic pipettes were used to produce wounds after cultured cells had reached 90% confluence. Cells were then washed twice with PBS and incubated in media containing 1% serum at 37 °C in a humidified incubator under 5% CO_2_. The wound closure was monitored over a 48- or 72-h period with a phase contrast microscope at ×200 magnification. For transwell assays, 4 × 10^4^ HCC cells were performed in transwell inserts with a 6.5-mm, 8.0-μm-pore polycarbonate membrane for migration assays, or Matrigel coated inserts for invasion assays (BD Biosciences). Cells were incubated for 48 or 72 h, and then non-migrating or non-invading cells on the inside of the membrane were carefully removed with a cotton swab, while migrating/invading cells on the outside of the membrane were fixed and stained with 0.5% Crystal violet in 70% ethanol, photographed under a light microscope. Five fields (200×) per membrane were randomly selected and then the average number of migrating or invading cells was determined.

### RNA isolation and quantitative real-time PCR

Total RNA from HCC cells and samples was extracted using an RNA Purification Kit (EZBioscience, Roseville, United States) and then was reverse- transcribed using an Advantage RT-for-PCR Kit (EZBioscience). The mRNA levels were determined and normalized against 18 s mRNA. qRT-PCR was performed with SYBR™ Green Universal Master Mix ExTaq (Thermo Fisher) with the Stratagene Mx3000P real-time PCR system (Agilent Technologies, Inc.). The sequences of primers are listed in Supplementary Table 1.

### Western blotting

Cells or liver tissues were harvested and frozen in liquid nitrogen. Protein extracts were prepared by using RIPA reagent kit (Beyotime, China) containing 1 mM PMSF (Beyotime, China). Equal amounts of protein were separated by SDS/ PAGE gels, transferred to Immobilon-P PVDF membranes (Millipore), and hybridized to an appropriate primary antibody and HRP-conjugated secondary antibody for subsequent detection by enhanced chemiluminescence (Amersham). The intensity of bands was analyzed by Image J. Primary antibodies used in this study were listed in Supplemental Table 2.

### Immunofluorescence

The cells were seeded on poly-lysine-coated chamber slides and subjected to starvation or treatment as indicated. The cell slides were fixed (4% paraformaldehyde), permeabilized (0.1% TritonX-100), blocked (10% Goat serum), and incubated with primary antibody in PBS overnight at 4 °C and with secondary antibody for 1 h at room temperature, counterstained with DAPI, mounted and visualized using a confocal microscope (LSM980, Leica, Wetzlar, Germany).

### Immunohistochemistry assay

Fixed tissue was prepared in 4% paraformaldehyde in phosphate-buffered saline (PBS; pH 7.0) and serially sectioned using paraffin using standard procedures. Before IHC staining, heat-induced epitope retrieval was conducted with retrieval buffer (EDTA pH 9.0 or citrate pH 6.0). Sections were incubated with primary antibodies listed in Supplementary Table 2. Haematoxylin-eosin (H&E) staining was performed for morphological examination.

### Molecular docking simulations

Rigid protein–protein docking (ZDOCK) was performed between SORT1 and p38, SORT1 and SREBP2 to study the relationships. Protein Data Bank PDB (http://www.rcsb.org/) was used to download the PDB format of the target protein structural domain. The ZDOCK module was run to identify the docking sites and calculate the ZDOCK scores.

### Co-IP

Co-IP was performed by following the manufacturer’s protocol (Thermo Fisher, catalog No. 26149). and immunoprecipitates were then subjected to Western blotting assay.

### Animal experiments

Male athymic nude mice (BALB/C-nu/nu, 4–5 weeks old) purchased from the animal center of Nanfang Hospital were used for xenograft studies. The mice were euthanized by cervical dislocation to prevent suffering. Nanfang Hospital Animal Ethics Committee approval (NFYY-2020-0952) was granted for this study.

Mice were intravenously injected with luciferase-labeled SMMC-7721 (2 × 106 cells/mouse) with different SORT1 expression levels to detect the effect of SORT1 on tumor metastasis in vivo. At 35 days post-caudal intravenous injection, all mice were sacrificed and dissected. Hematoxylin and eosin staining were used to detect metastatic nodules in the lungs. A bioluminescent IVIS 100 Imaging System (Xenogen, CA, USA) was used to confirm the presence of lung metastases in these mice. When tumors reached an appropriate size, animals were euthanized, and tumor tissues were excised for IHC analyses.

### Statistical analysis

All experiments were independently performed three times unless otherwise stated. Data are presented as mean ± standard deviation (s.d.). Pearson correlation coefficients were used to evaluate the relationships between SORT1 and p38, SREBP2 expression. Statistical significance was determined by Student’s *t*-test, one-way ANOVA, Log-rank test, or Fisher’s exact test. For all statistical tests, the 0.05 level of confidence (two-sided) was accepted for statistical significance

## Supplementary information

Supplementary Figure Legends and Tables

Supplementary Figure 1

Supplementary Figure 2

Supplementary Figure 3

Uncropped western blots

## Data Availability

The data that support the findings of this study are available from the corresponding author, SHC, upon reasonable request.
