# Supplementary material for: SORT1 promote the metastasis and invasion of hepatocellular carcinoma via p38/β-catenin/ZEB1 signaling pathway
Source: Cell Death Dis. 2025 Aug 1;16(1):582. doi: 10.1038/s41419-025-07871-y (PMC12317026; doi:10.1038/s41419-025-07871-y)
Supplement: Supplementary file 1 — Supplementary Figure Legends and Tables [file 41419_2025_7871_MOESM1_ESM.docx]

**Supplementary figure 1:** SORT1 does not influence proliferation of HCC cells.

(A) The proliferative capacity of SORT1-depleted SMMC-7721 and Huh-7 cells was assessed by MTT. (B) EdU assays were performed to assess the proliferative ability of SMMC-7721 and Huh-7 cells with SORT1 knockdown. (C) The cell cycle of SORT1-depleted SMMC-7721 and Huh-7 cells were analyzed by PI staining using flow cytometry.

**Supplementary figure 2:** SORT1 promote exosome and exosomal MMP9 protein secretion via Golgi.

(A) Diameter distribution analysis of Huh-7 cell exosomes. (B) Quantification of exosome diameter. (C) Scanning electron microscope image of Huh-7 cell exosomes. (D) Protein extracts from exosomes were assessed by western blot analysis using antibodies against exosomal protein markers (TSG101, CD9, Grp94 and Alix). (E) MMP9 and β-actin were detected in exosomal protein extracting from SMMC-7721 and Huh-7 cell. (F) Wound-healing assays measuring cell motility in SMMC-7721 and Huh-7 cells culturing with exosome from control or SORT1-overexpression cell. (G) Immunofluorescence staining was used to determine the length of Golgi in SMMC-7721 and Huh-7 cell. (H) Exosomal MMP9 levels in HCC patients was compared to healthy controls from the ExoRBase exosome database. Quantitative data are presented as the mean±SD. *p< 0.05; **p < 0.01; ***p < 0.001.

**Supplementary figure 3:** Supporting data of SREBP2/SORT1/p38/β-catenin/ZEB1 signaling axis.

1. ERK, JNK and AKT pathways in SORT1-knockdown HCC cells were measured by western blot. Correlations between SORT1 and ZEB1 expression in HCC cohorts from TIMER (B) and GEPIA (D) database. (C) β-catenin nuclear expression was measure in rescue (SORT1-knockdown and p38 overexpression) SMMC-7721 and Huh-7 HCC cells model. Correlations between SREBP2 and SORT1 expression in HCC cohorts from TIMER (F) and GEPIA (H) database. (E) Molecular docking of SORT1 into the p38. The ZDOCK Score was 1235.845. (G) Molecular docking of SREBP2 into the SORT1. The ZDOCK Score was 1314.695.

Supplementary Table 1. Sequences for siRNA and primers used in this study.

| Name | Sequence |
| --- | --- |
| siSORT1-1 | 5’-AAUUCAGUCCGAAUAAAGGTG-3’ |
| siSORT1-2 | 5’-AUACCAUGUCAUCAUUUGCTG-3’ |
| siZEB1-1 | 5’-GGTAGATGGTAATGTAATAT-3’ |
| siSREBP2-1 | 5’-AUCUGUCUUGAUGAUCUGAGGTT-3’ |
| siSREBP2-1 | 5’-UUCAGGAGAGAGAAACUCAGGTT-3’ |
| SORT1-Forward | 5’-GAAGTCGTGGAGGAAGAATCTTT-3’ |
| SORT1-Reverse | 5’-TGGTGTTGTCTGATCCCCATT-3’ |
| SREBP2-Forward | 5’-CCTGGGAGACATCGACGAGAT-3’ |
| SREBP2-Reverse | 5’-TGAATGACCGTTGCACTGAAG-3’ |
| ZEB1-Forward | 5’-GATGATGAATGCGAGTCAGATGC-3’ |
| ZEB1-Reverse | 5’-ACAGCAGTGTCTTGTTGTTGT-3’ |
| C-myc-Forward | GGCTCCTGGCAAAAGGTCA-3’ |
| C-myc-Reverse | CTGCGTAGTTGTGCTGATGT-3’ |
| cyclin-D1-Forward | CAGAGGCGGAGGAGAACAAA-3’ |
| cyclin-D1-Reverse | ATGGAGGGCGGATTGGAA-3’ |
| MMP9-Forward | 5’-TGTACCGCTATGGTTACACTCG-3’ |
| MMP9-Reverse | 5’-GGCAGGGACAGTTGCTTCT-3’ |
| MMP7-Forward | GAGTGAGCTACAGTGGGAACA-3’ |
| MMP7-Reverse | CTATGACGCGGGAGTTTAACAT-3’ |
| GAPDH-Forward | 5’-ACAACTTTGGTATCGTGGAAGG-3’ |
| GAPDH-Reverse | 5’-GCCATCACGCCACAGTTTC-3’ |

Supplementary Table 2. Antibodies used in this study.

| Name | Dilusion | Catalogue | Company |
| --- | --- | --- | --- |
| SORT1 | 1/1000 | ab263864 | abcam |
| SREBP2 | 1/1000 | ab112046 | abcam |
| p38 | 1/1000 | AF6456 | Affinity |
| Phospho-p38 | 1/1000 | AF4001 | Affinity |
| ZEB1 | 1/1000 | DF7414 | Affinity |
| β-catenin | 1/1000 | AF6266 | Affinity |
| Phospho-β-catenin | 1/1000 | DF2989 | Affinity |
| GFP | 1/1000 | ab127417 | abcam |
| GOLGA5 | 1/400 | H00051108-B01P | Novus |
| GSK3β | 1/1000 | [AF5016](http://www.affbiotech.cn/goods-1681-AF5016-GSK3_beta_Antibody.html) | Affinity |
| Phospho-GSK3β(Ser9) | 1/1000 | [AF2016](http://www.affbiotech.cn/goods-1185-AF2016-Phospho_GSK3_beta_Ser9_Antibody.html) | abcam |
| E-cadherin | 1/1000 | 3195T | CST |
| N-cadherin | 1/1000 | 13116T | CST |
| Vimentin | 1/1000 | 5741T | CST |
| MMP9 | 1/1000 | AF5228 | Affinity |
| β-actin | 1/1000 | Ab8227 | abcam |
| β-catenin | 1/100 | 8480 | CST |
| ERK | 1/1000 | BF8004 | Affinity |
| Phospho-ERK | 1/1000 | AF1015 | Affinity |
| AKT | 1/1000 | AF4718 | Affinity |
| Phospho-AKT | 1/1000 | AF0016 | Affinity |
| JNK | 1/1000 | AF6318 | Affinity |
| Phospho-JNK | 1/1000 | AF3319 | Affinity |
| c-jun | 1/1000 | AF6090 | Affinity |
| Phospho-c-jun | 1/1000 | AF3095 | Affinity |
| Lamin B1 | 1/1000 | AF5161 | Affinity |

Supplementary Table 3. Correlation of SORT1 expression and clinical features of patients with HCC.

| Variable | SORT1 expression | | | |
| --- | --- | --- | --- | --- |
|  | All case | Low expression | High expression | P value^a^ |
| Age (years) |  |  |  | 0.401 |
| ≤ 49 | 374 | 215 | 159 |  |
| > 49 | 407 | 246 | 161 |  |
| Gender |  |  |  | 0.798 |
| Male | 691 | 409 | 282 |  |
| Female | 90 | 52 | 38 |  |
| HBV |  |  |  | 0.110 |
| Negative | 135 | 88 | 47 |  |
| Positive | 646 | 373 | 273 |  |
| AFP (ng/ml) |  |  |  | 0.120 |
| ≤20 | 173 | 111 | 62 |  |
| >20 | 608 | 350 | 258 |  |
| Cirrhosis |  |  |  | 0.877 |
| Yes | 639 | 378 | 261 |  |
| No | 142 | 83 | 59 |  |
| Tumor size (cm) |  |  |  | 0.491 |
| ≤ 5 | 193 | 118 | 75 |  |
| > 5 | 588 | 343 | 245 |  |
| Tumor multiplicity |  |  |  | 0.039 |
| Single | 516 | 318 | 198 |  |
| Multiple | 265 | 143 | 122 |  |
| Differentiation |  |  |  | 0.436 |
| Well-Moderate | 64 | 41 | 23 |  |
| Poor-Undifferentiated | 711 | 420 | 291 |  |
| Stage |  |  |  | <0.001 |
| I-II | 323 | 242 | 81 |  |
| III-IV | 458 | 219 | 239 |  |
| Tumor capsule |  |  |  | 0.811 |
| Absent | 450 | 264 | 186 |  |
| Present | 331 | 197 | 134 |  |
| LNM |  |  |  | 0.339 |
| Yes | 44 | 29 | 15 |  |
| No | 737 | 432 | 305 |  |
| Vascular invasion |  |  |  | <0.001 |
| Yes | 144 | 65 | 79 |  |
| No | 637 | 396 | 241 |  |

a Chi-square test; b Median age; AFP, alpha-fetoprotein; HBsAg, hepatitis B surface antigen; LNM,

lymph node metastasis.

Supplementary Table 4. Univariate and multivariate analysis of high SORT1 expression and overall survival.

| Variable | Univariate analysis | | Multivariate analysis | |
| --- | --- | --- | --- | --- |
|  | HR (95% CI) | P value | HR (95% CI) | P value |
| Age (≤ 49 vs. > 49 years) | 0.869 (0.747-1.010) | 0.067 |  |  |
| Gender (female vs. male) | 0.866 (0.679-1.104) | 0.246 |  |  |
| HBV (positive vs. negative) | 1.171 (0.953-1.439) | 0.133 |  |  |
| Tumor size (≤ 5 vs. > 5 cm) | 1.650 (1.375-1.980) | **<0.001** | 1.449 (1.197-1.753) | **<0.001** |
| Tumor multiplicity (single vs. multiple) | 1.634 (1.395-1.914) | **<0.001** | 1.155 (0.957-1.393) | 0.133 |
| Tumor capsule | 1.360 (1.166-1.588) | **<0.001** | 1.151 (0.978-1.355) | 0.091 |
| Liver cirrhosis (yes vs. no) | 0.979 (0.803-1.194) | 0.836 |  |  |
| AFP (≤ 20 vs. > 20 ng/mL) | 1.260 (1.052-1.508) | **0.012** | 1.062 (0.877-1.285) | 0.538 |
| LNM (Yes vs. no) | 1.902 (1.395-2.594) | **<0.001** | 1.528 (1.102-2.118) | **0.011** |
| Tumor differentiation | 1.635 (1.245-2.146) | **<0.001** | 1.303 (0.976-1.741) | 0.073 |
| TNM (I-II vs. III-IV) | 2.067 (1.765-2.420) | **<0.001** | 1.683 (1.417-1.999) | **<0.001** |
| Vascular invasion (Yes vs. No) | 2.575 (2.131-3.111) | **<0.001** | 1.737 (1.407-2.143) | **<0.001** |
| SORT1 expression (high vs. low) | 1.174 (1.005-1.371) | **0.043** | 0.916 (0.778-1.079) | 0.294 |

HR, hazard ratio; CI, confident interval; AFP, alpha-fetoprotein; HBsAg, hepatitis B surface antigen;

LNM, lymph node metastasis

Supplementary Table 5. Univariate and multivariate analysis of high SORT1 expression and disease-free survival.

| Variable | Univariate analysis | | Multivariate analysis | |
| --- | --- | --- | --- | --- |
|  | HR (95% CI) | P value | HR (95% CI) | P value |
| Age (≤ 49 vs. > 49 years) | 0.808 (0.670-0.997) | **0.047** | 0.847 (0.692-1.037) | 0.108 |
| Gender (female vs. male) | 0.875 (.0635-1.206) | 0.414 |  |  |
| HBV (positive vs. negative) | 0.992 (0.763-1.289) | 0.951 |  |  |
| Tumor size (≤ 5 vs. > 5 cm) | 1.224 (0.976-1.534) | **0.080** | 1.148 (0.910-1.449) | 0.244 |
| Tumor multiplicity (single vs. multiple) | 1.200(0.968-1.488) | **0.097** | 1.126 (0.867-1.462) | 0.373 |
| Tumor capsule | 1.170 (0.957-1.431) | 0.126 |  |  |
| Liver cirrhosis (yes vs. no) | 1.013 (0.784-1.308) | 0.921 |  |  |
| AFP (≤ 20 vs. > 20 ng/mL) | 1.281 (1.007-1.630) | **0.044** | 1.193 (0.932-1.527) | 0.162 |
| LNM (Yes vs. no) | 0.921 (0.558-1.520) | 0.747 |  |  |
| Tumor differentiation | 1.294 (0.925-1.811) | 0.132 |  |  |
| TNM (I-II vs. III-IV) | 1.225 (1.002-1.499) | **0.048** | 0.895 (0.706-1.135) | 0.360 |
| Vascular invasion (Yes vs. No) | 1.555 (1.195-2.025) | **0.001** | 1.402 (1.067-1.841) | **0.015** |
| SORT1 expression (high vs. low) | 1.710 (1.396-2.094) | **<0.001** | 1.661 (1.354-2.038) | **<0.001** |

HR, hazard ratio; CI, confident interval; AFP, alpha-fetoprotein; HBsAg, hepatitis B surface antigen;

LNM, lymph node metastasis

Supplementary Table 6. Univariate and multivariate analysis of high SORT1 expression and tumor recurrence.

| Variable | Univariate analysis | | Multivariate analysis | |
| --- | --- | --- | --- | --- |
|  | OR (95% CI) | P value | OR (95% CI) | P value |
| Age (≤ 49 vs. > 49 years) | 0.993 (0.752-1.312) | 0.962 |  |  |
| Gender (female vs. male) | 0.789 (0.508-1.226) | 0.292 |  |  |
| HBV (positive vs. negative) | 1.006 (0.693-1.461) | 0.975 |  |  |
| Tumor size (≤ 5 vs. > 5 cm) | 0.648 (0.469-0.894) | **0.008** | 0.681 (0.485-0.954) | **0.026** |
| Tumor multiplicity (single vs. multiple) | 0.814 (0.606-1.093) | 0.172 |  |  |
| Tumor capsule | 0.793 (0.599-1.051) | 0.107 |  |  |
| Liver cirrhosis (yes vs. no) | 0.979 (0.685-1.398) | 0.906 |  |  |
| AFP (≤ 20 vs. > 20 ng/mL) | 1.011 (0.724-1.412) | 0.949 |  |  |
| LNM (Yes vs. no) | 0.464 (0.240-0.896) | **0.022** | 0.587 (0.296-1.166) | 0.128 |
| Tumor differentiation | 0.600 (0.365-0.987) | **0.044** | 0.712 (0.416-1.217) | 0.214 |
| TNM (I-II vs. III-IV) | 0.695 (0.524-0.922) | **0.012** | 0.576 (0.421-0.790) | **0.001** |
| Vascular invasion (Yes vs. No) | 0.756 (0.526-1.085) | 0.129 |  |  |
| SORT1 expression (high vs. low) | 2.011 (1.505-2.688) | **<0.001** | 2.408 (1.764-3.287) | **<0.001** |

HR, hazard ratio; CI, confident interval; AFP, alpha-fetoprotein; HBsAg, hepatitis B surface antigen;

LNM, lymph node metastasis

Supplementary Table 7. Correlation of SREEBP2 expression and clinical features of patients with HCC.

| Variable | SREEBP2 expression | | | |
| --- | --- | --- | --- | --- |
|  | All case | Low expression | High expression | P value^a^ |
| Age (years) |  |  |  | 0.448 |
| ≤ 49 | 374 | 272 | 102 |  |
| > 49 | 407 | 286 | 121 |  |
| Gender |  |  |  | 0.286 |
| Male | 691 | 498 | 193 |  |
| Female | 90 | 60 | 30 |  |
| HBV |  |  |  | 0.582 |
| Negative | 131 | 96 | 35 |  |
| Positive | 646 | 458 | 188 |  |
| AFP (ng/ml) |  |  |  | 0.760 |
| ≤20 | 173 | 122 | 51 |  |
| >20 | 608 | 436 | 172 |  |
| Cirrhosis |  |  |  | 0.052 |
| Yes | 639 | 466 | 173 |  |
| No | 142 | 92 | 50 |  |
| Tumor size (cm) |  |  |  | 0.369 |
| ≤ 5 | 193 | 133 | 60 |  |
| > 5 | 588 | 425 | 163 |  |
| Tumor multiplicity |  |  |  | 0.911 |
| Single | 516 | 368 | 148 |  |
| Multiple | 265 | 190 | 75 |  |
| Differentiation |  |  |  | 0.730 |
| Well-Moderate | 64 | 47 | 17 |  |
| Poor-Undifferentiated | 711 | 507 | 203 |  |
| Stage |  |  |  | **0.049** |
| I-II | 323 | 243 | 80 |  |
| III-IV | 458 | 315 | 143 |  |
| Tumor capsule |  |  |  | 0.180 |
| Absent | 450 | 331 | 119 |  |
| Present | 331 | 229 | 102 |  |
| LNM |  |  |  | 0.591 |
| Yes | 44 | 33 | 11 |  |
| No | 737 | 525 | 212 |  |
| Vascular invasion |  |  |  | 0.400 |
| Yes | 144 | 107 | 37 |  |
| No | 637 | 451 | 186 |  |

a Chi-square test; b Median age; AFP, alpha-fetoprotein; HBsAg, hepatitis B surface antigen; LNM,

lymph node metastasis.

Supplementary Table 8. List of proteins identified in co-immunoprecipitated SORT1 complex by MS/MS.

| Gene Name | Score | Coverage |
| --- | --- | --- |
| HEL113 | 7855 | 84% |
| PLEC | 6423 | 40% |
| MYO1C | 3983 | 64% |
| ACTG1 | 3496 | 76% |
| MYO1C | 3204 | 63% |
| SREBP2 | 1954 | 58% |
| TUBB2C | 1882 | 70% |
| FLJ00293 | 1795 | 60% |
| MYO1G | 1776 | 51% |
| SPTBN1 | 1698 | 35% |
| ACTN4 | 1461 | 60% |
| TUBA1C | 1329 | 57% |
| DBN1 | 1314 | 53% |
| HEL-S-89n | 1303 | 49% |
| TUBA4A | 1166 | 48% |
| LIMA1 | 1078 | 49% |
| MYH9 | 883 | 19% |
