# Supplementary figures and images for: SORT1 promote the metastasis and invasion of hepatocellular carcinoma via p38/β-catenin/ZEB1 signaling pathway
Source: Cell Death Dis. 2025 Aug 1;16(1):582. doi: 10.1038/s41419-025-07871-y (PMC12317026; doi:10.1038/s41419-025-07871-y)

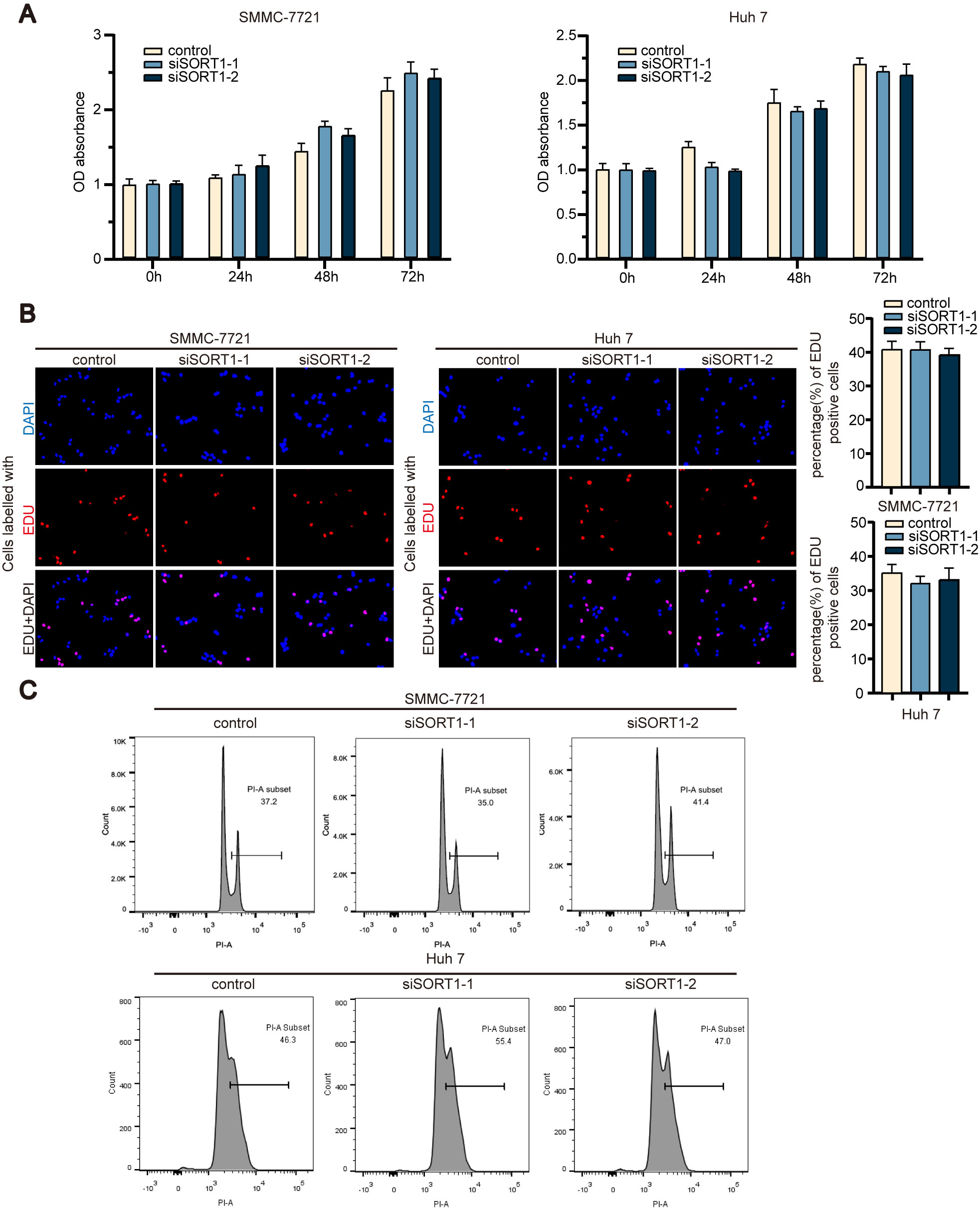

Supplement: Supplementary file 2 — Supplementary Figure 1 [file 41419_2025_7871_MOESM2_ESM.jpg]

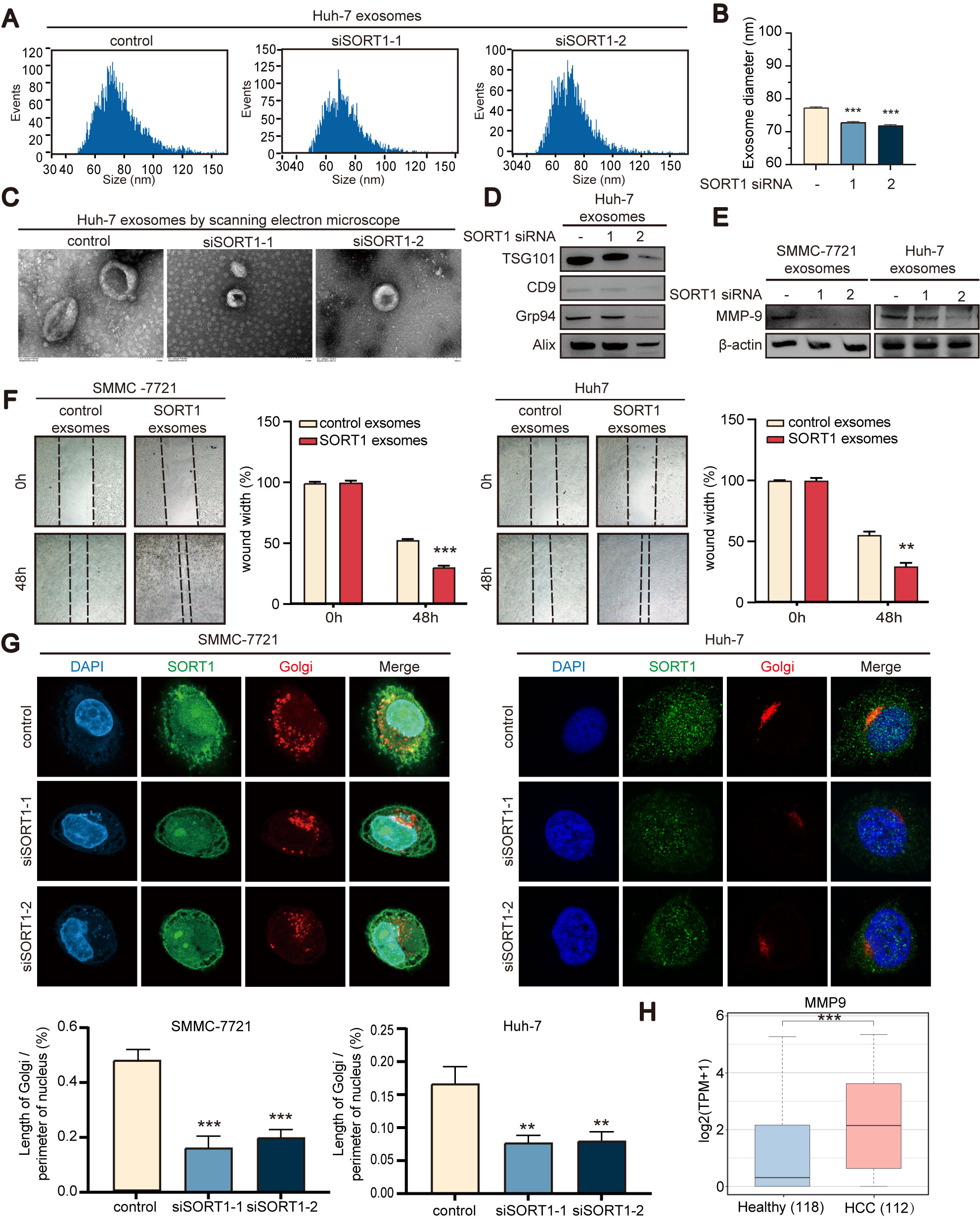

Supplement: Supplementary file 3 — Supplementary Figure 2 [file 41419_2025_7871_MOESM3_ESM.jpg]

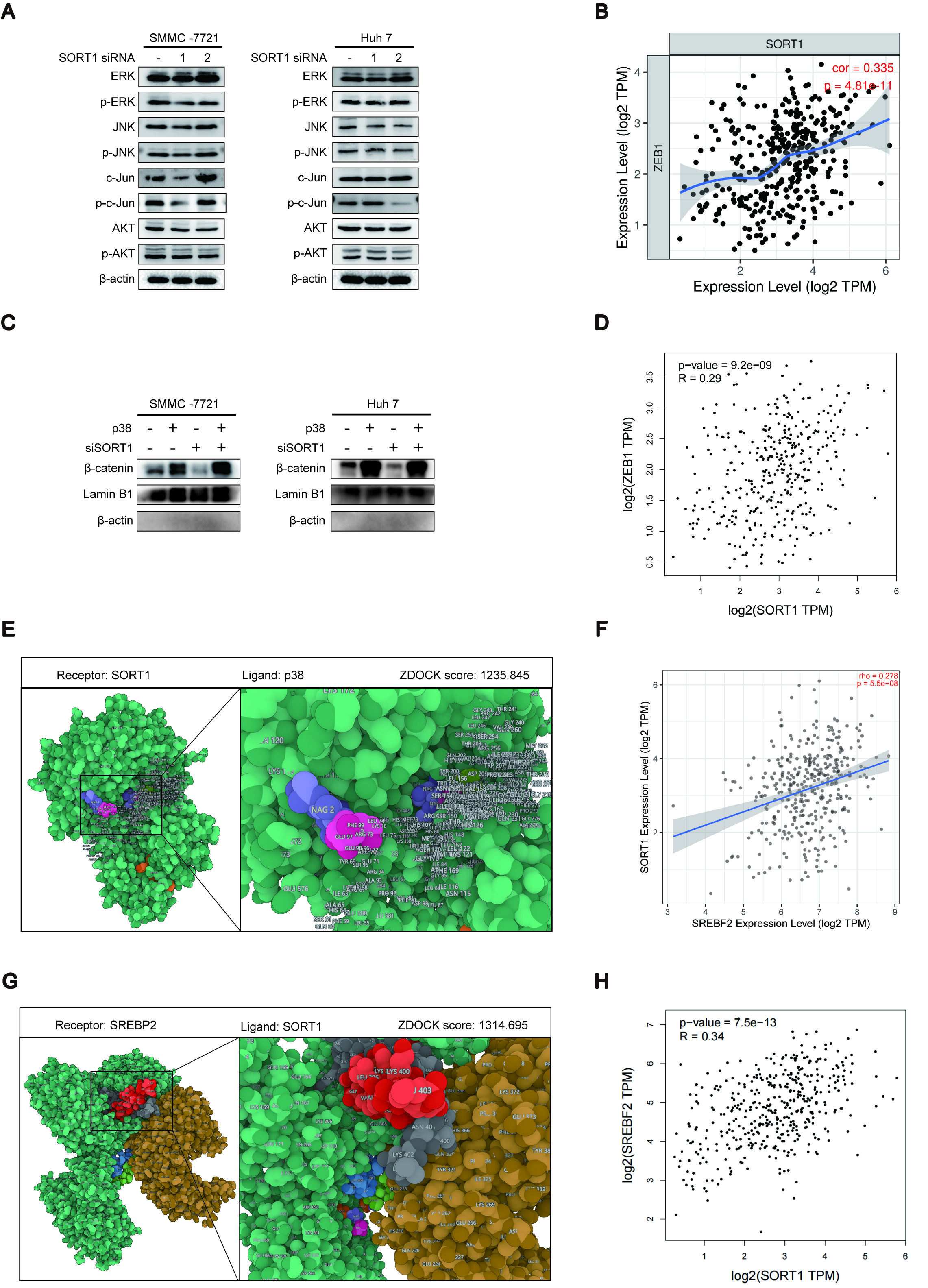

Supplement: Supplementary file 4 — Supplementary Figure 3 [file 41419_2025_7871_MOESM4_ESM.jpg]
