# Supplementary material for: SORT1 promote the metastasis and invasion of hepatocellular carcinoma via p38/β-catenin/ZEB1 signaling pathway
Source: Cell Death Dis. 2025 Aug 1;16(1):582. doi: 10.1038/s41419-025-07871-y (PMC12317026; doi:10.1038/s41419-025-07871-y)

Figure 1B

SORT1

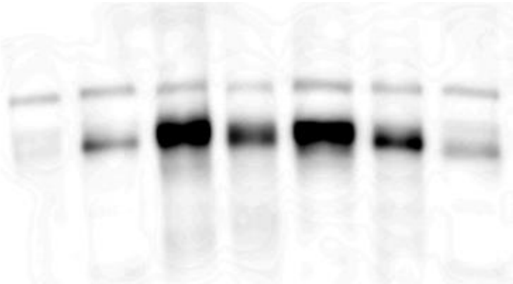

β-actin

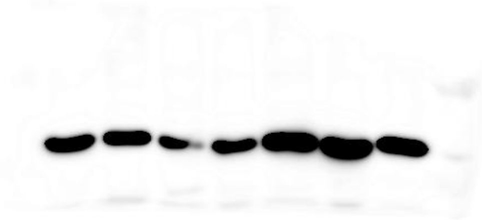

Figure 1D

SORT1

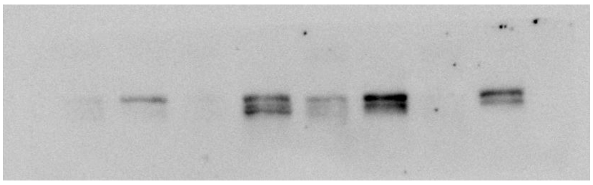

β-actin

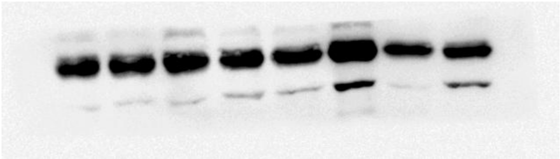

Figure 2A

SORT1

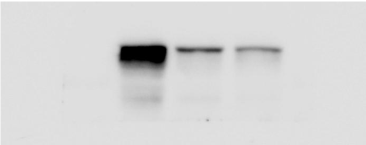

SORT1

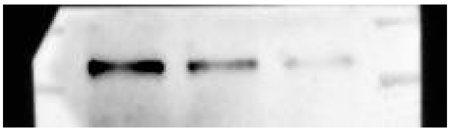

β-actin

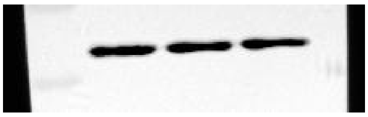

β-actin

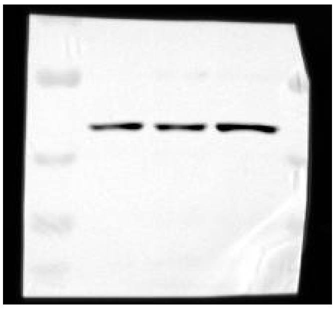

Figure 2B

SORT1

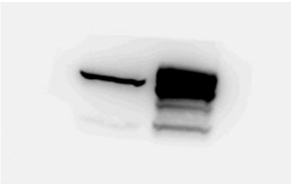

SORT1

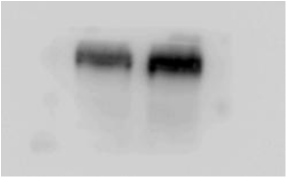

β-actin

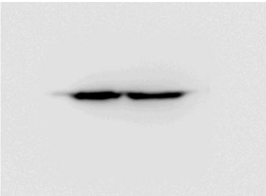

β-actin

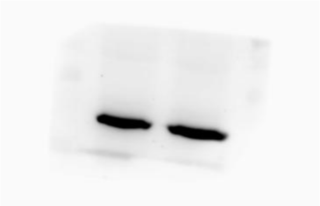

Figure 3B

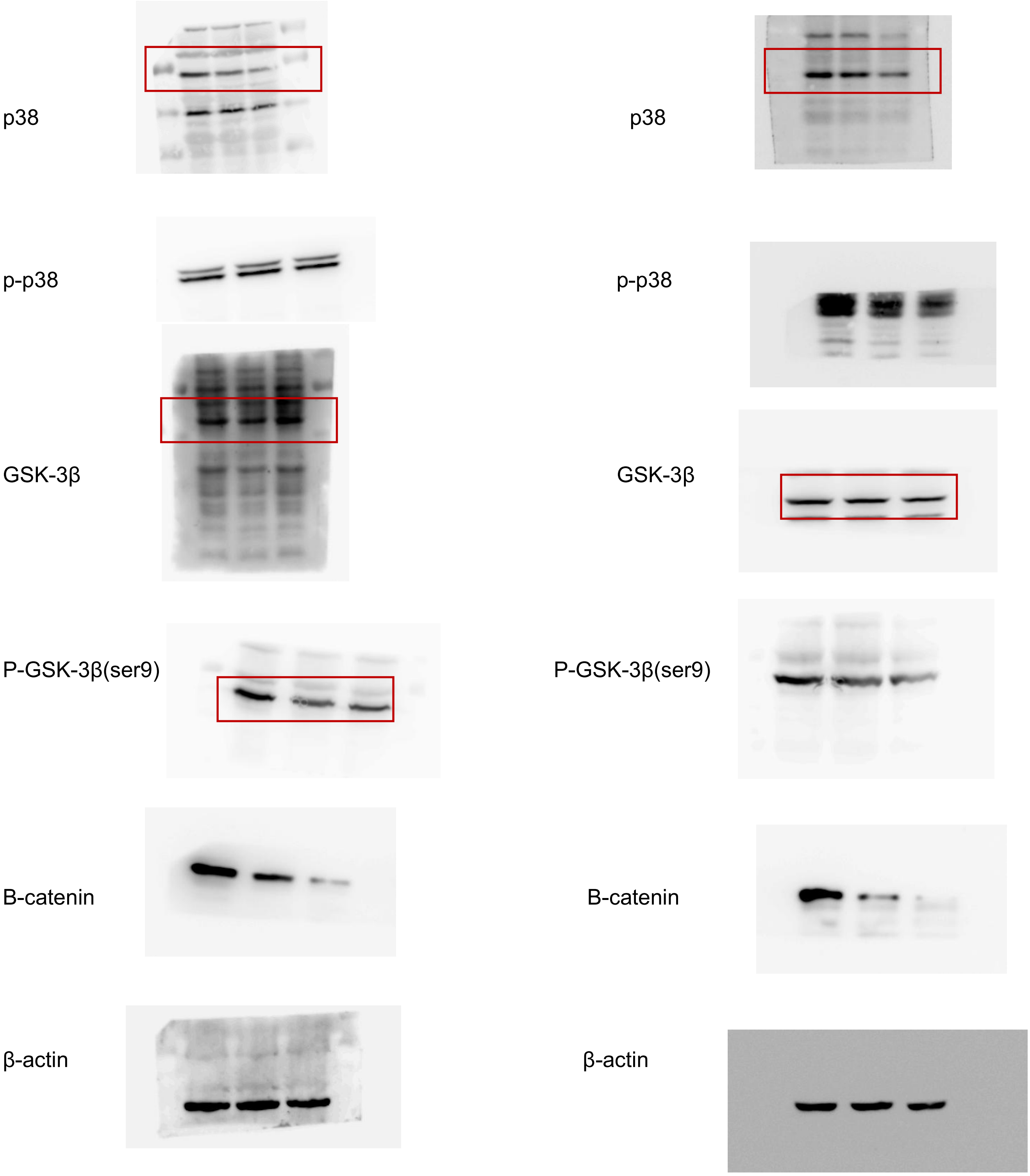
p38
p38
p-p38
p-p38
GSK-3β
GSK-3β
P-GSK-3β(ser9)
P-GSK-3β(ser9)
B-catenin
B-catenin
β-actin
β-actin

Figure 3C

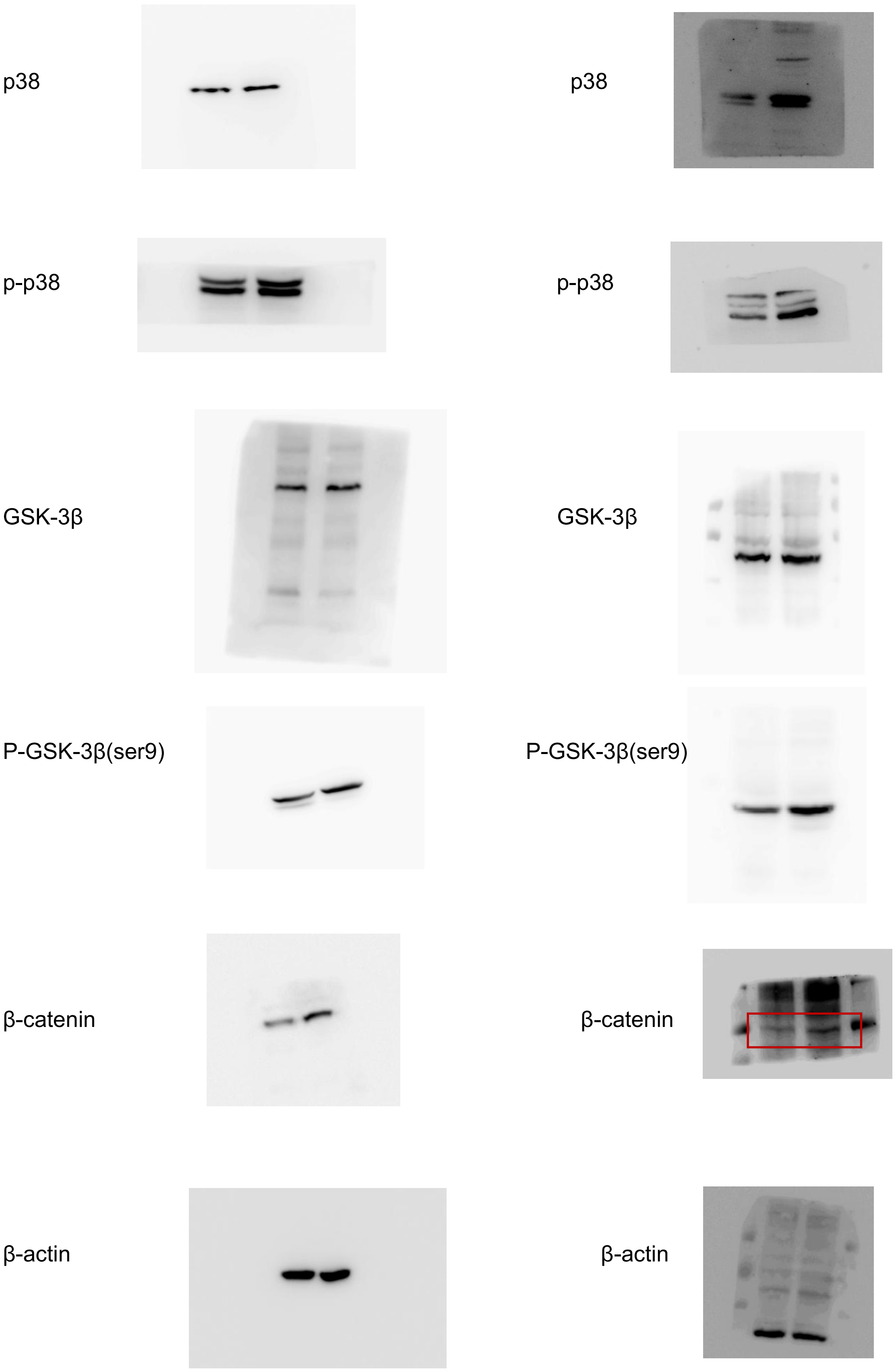
p38
p38
p-p38
p-p38
GSK-3β
GSK-3β
P-GSK-3β(ser9)
P-GSK-3β(ser9)
β-catenin
β-catenin
β-actin
β-actin

Figure 3D

P38

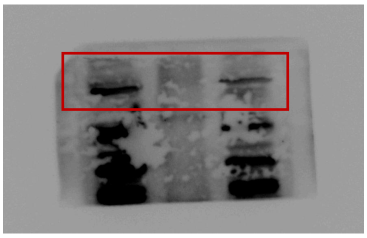

P38

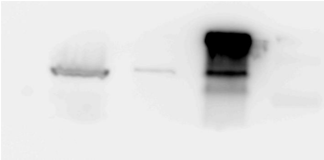

GFP

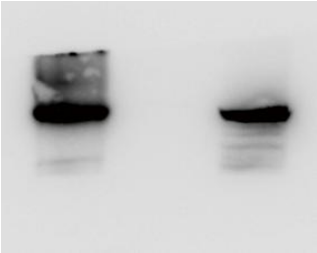

GFP

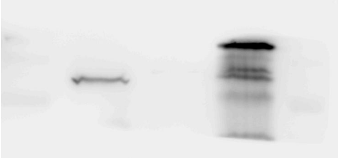

P38

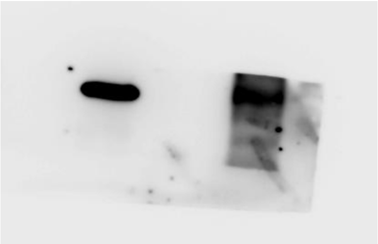

P38

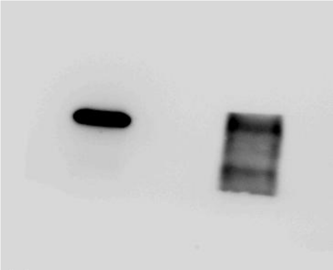

GFP

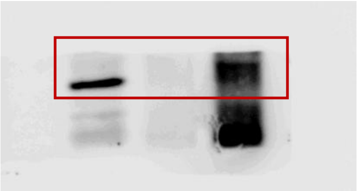

GFP

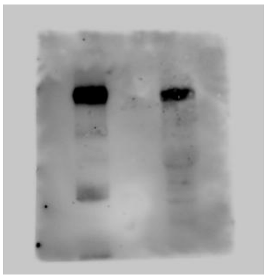

Figure 3E

p38

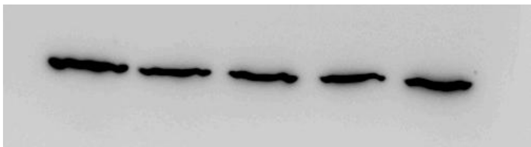

p38

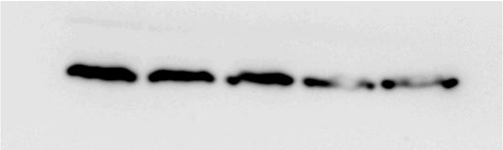

β-actin

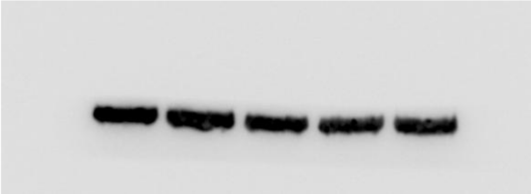

β-actin

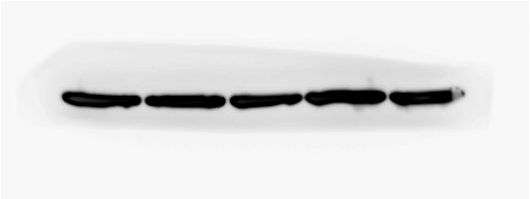

p38

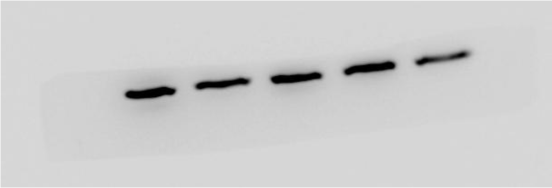

p38

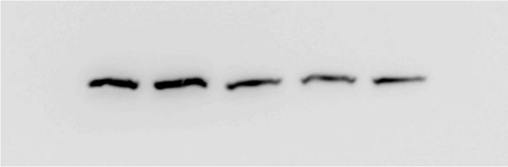

β-actin

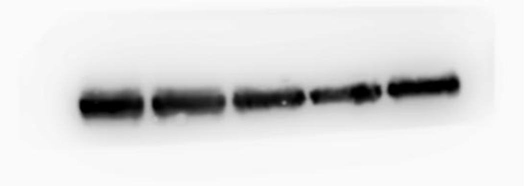

β-actin

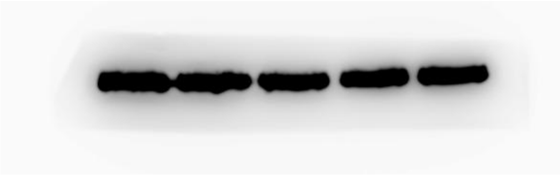

Figure 4C

ZEB1

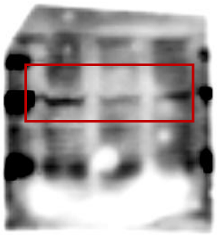

ZEB1

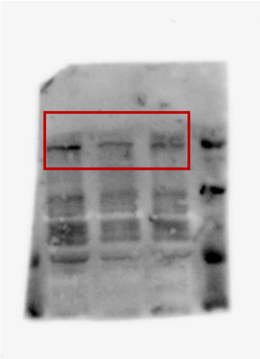

β-actin

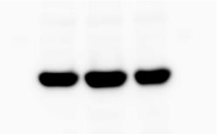

β-actin

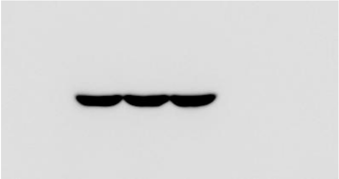

ZEB1

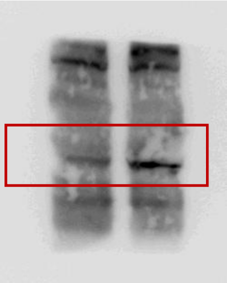

ZEB1

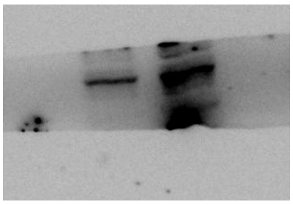

β-actin

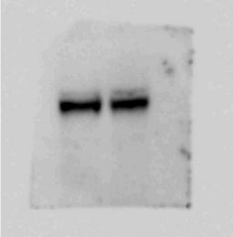

β-actin

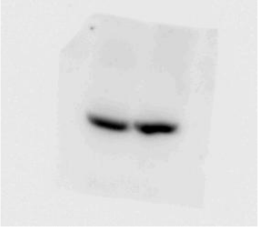

Figure 6B

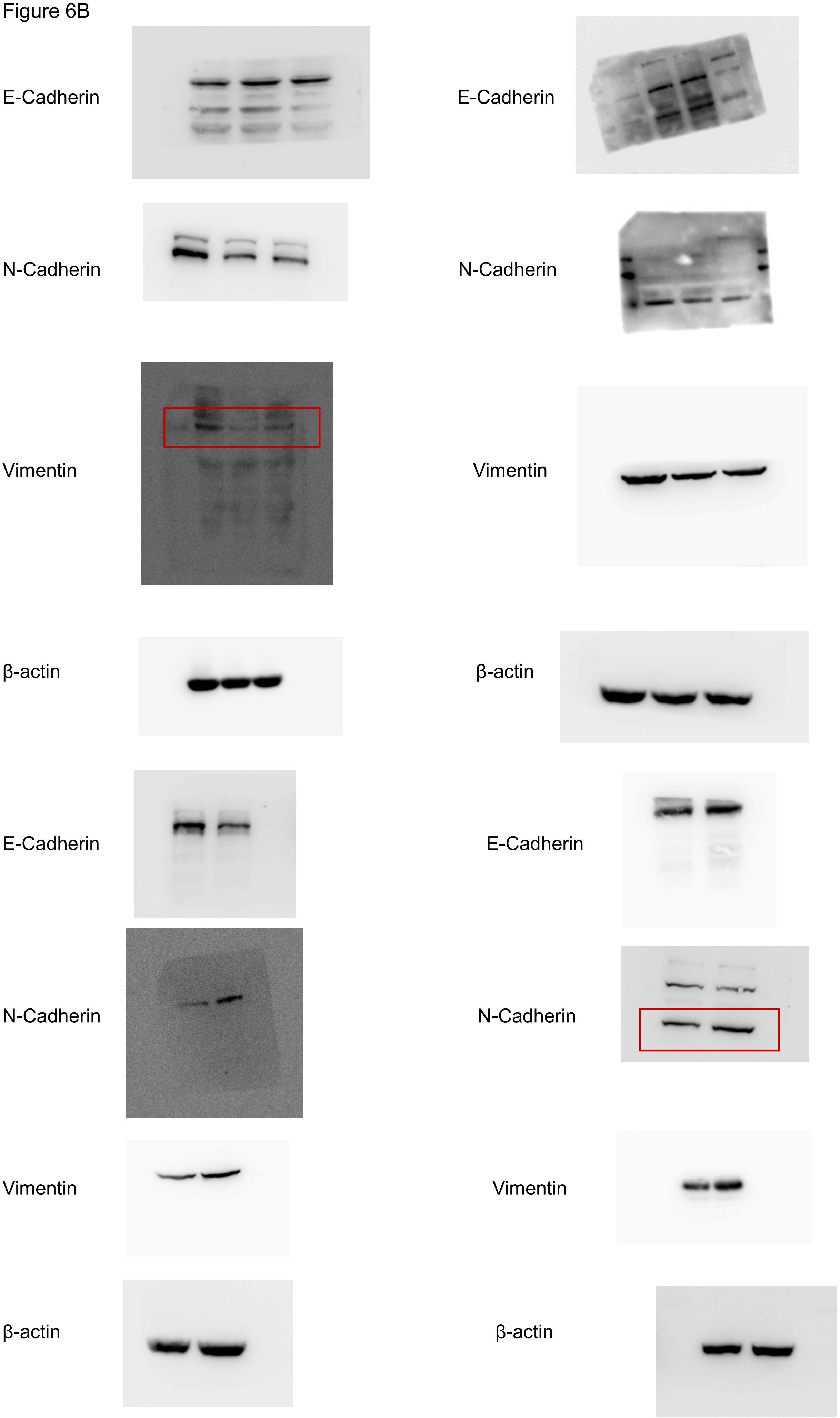
E-Cadherin
E-Cadherin
N-Cadherin
N-Cadherin
Vimentin
Vimentin
β-actin
β-actin
E-Cadherin
E-Cadherin
N-Cadherin
N-Cadherin
Vimentin
Vimentin
β-actin
β-actin

Figure 7C

SORT1

β-actin

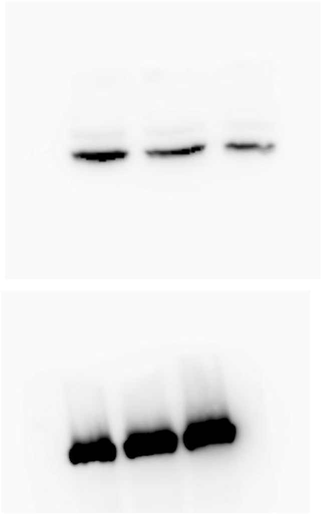

Figure 7F

SORT1

β-actin

SORT1

β-actin

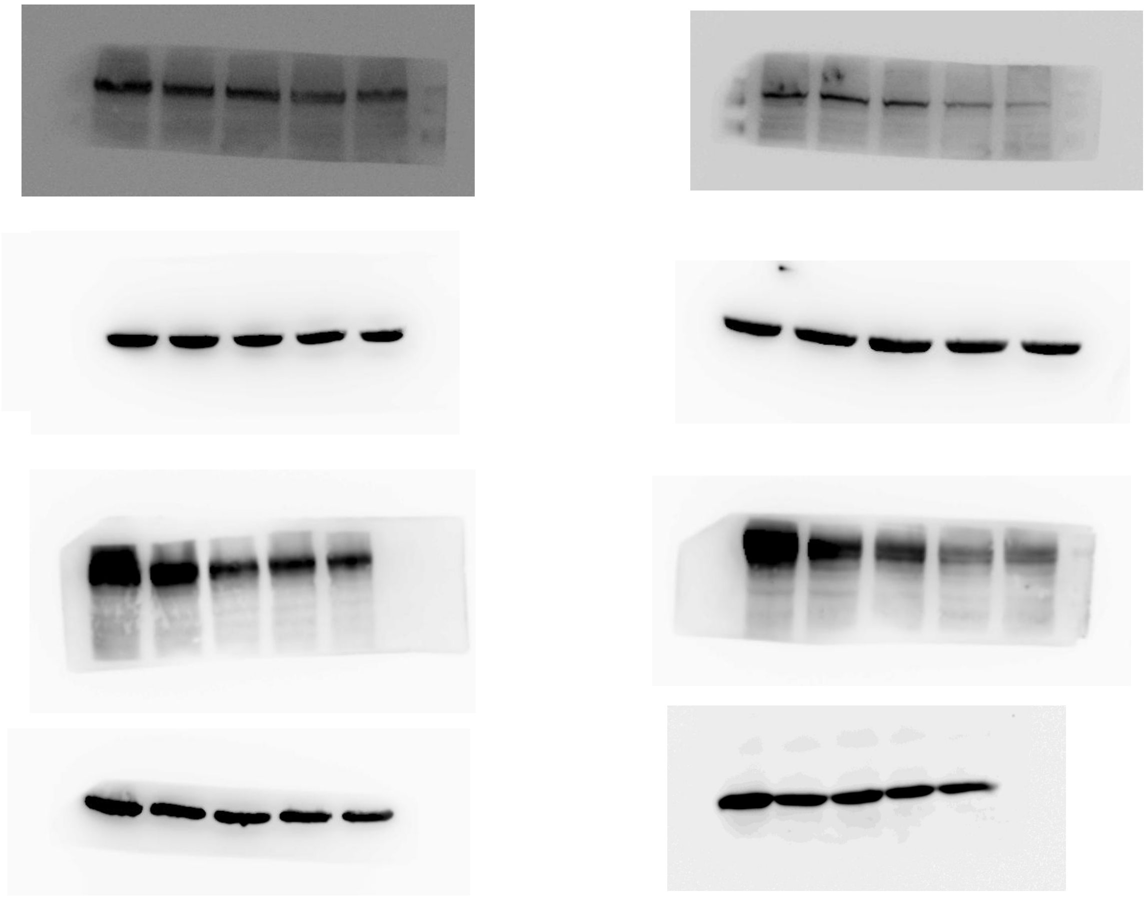

Figure 7G

SORT1

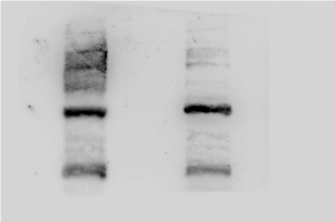

SREBP2

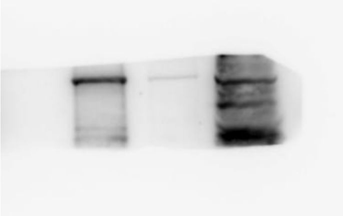

Supplementary Figure 2B

TSG101

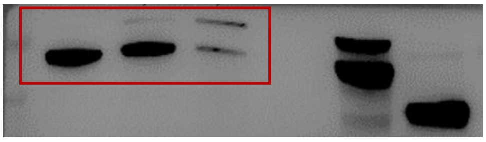

CD9

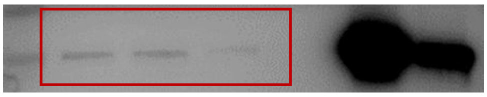

Grp94

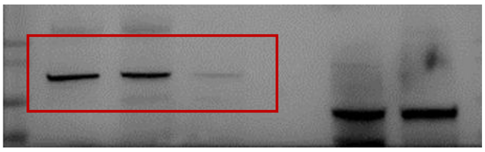

Alix

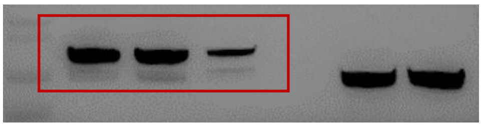

Supplementary Figure 2D

MMP9

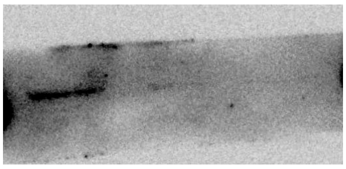

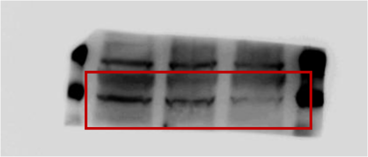

β-actin

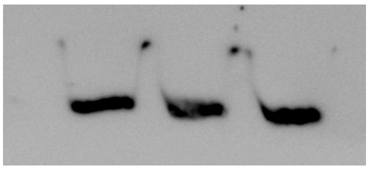

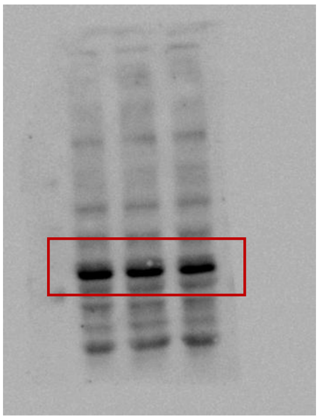

Supplementary Figure 3A

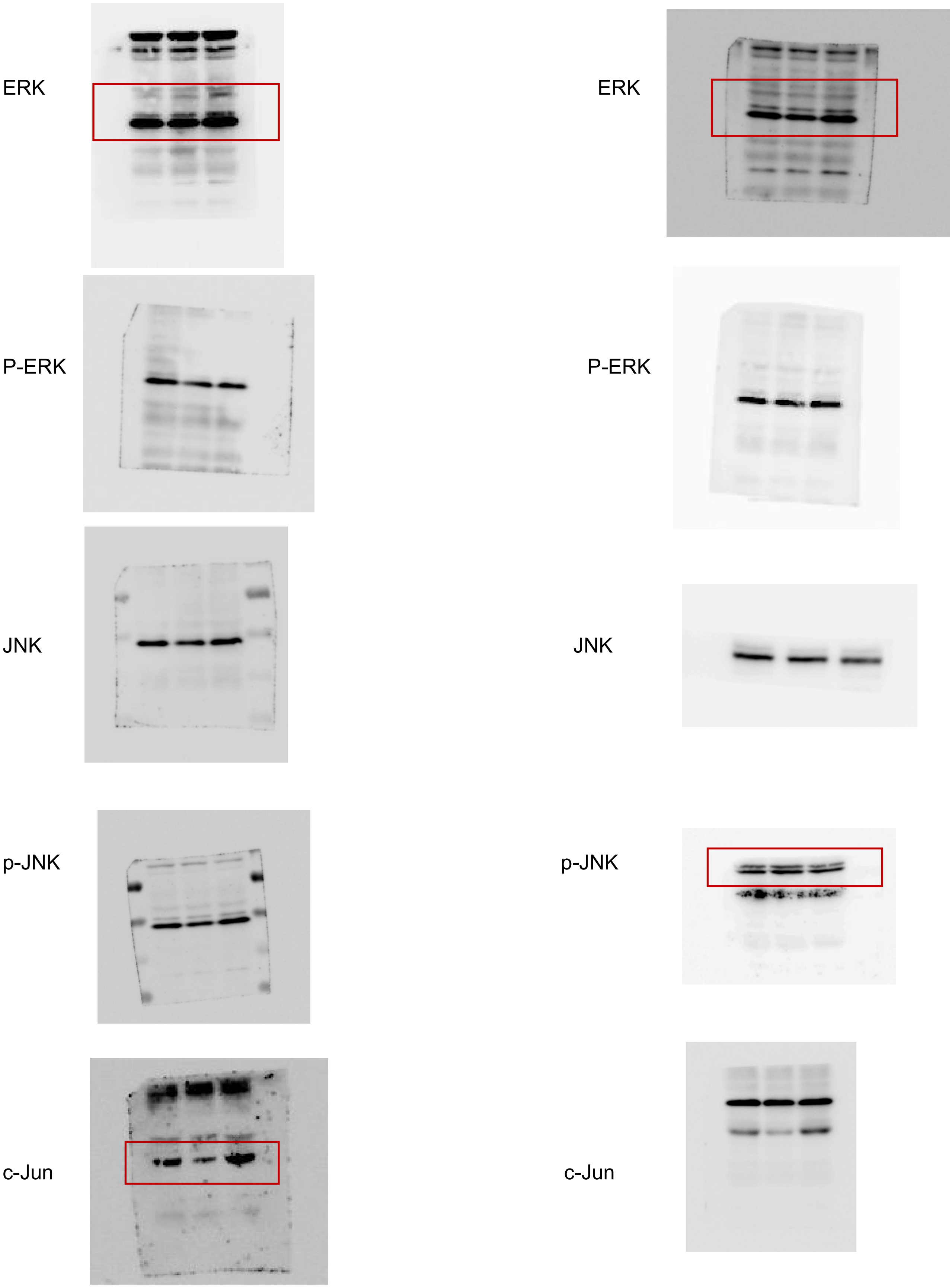

p-c-Jun

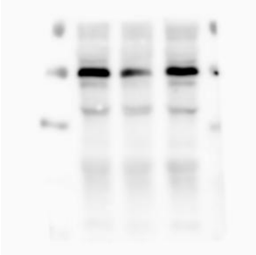

p-c-Jun

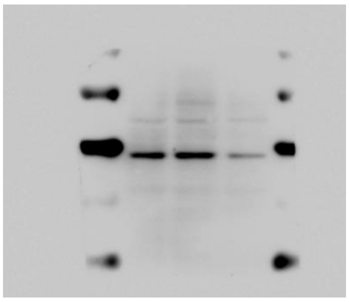

AKT

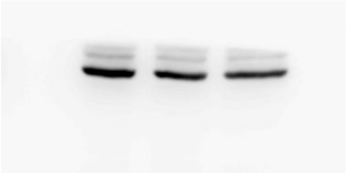

AKT

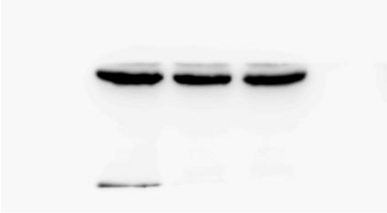

p-AKT

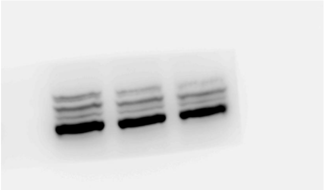

p-AKT

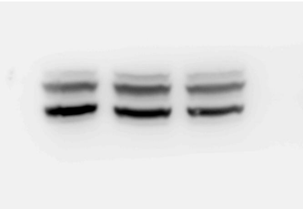

β-actin

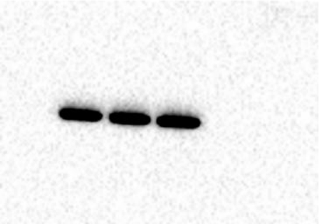

β-actin

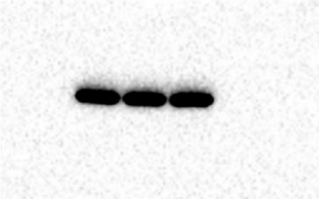

Supplementary Figure 3C

B-catenin

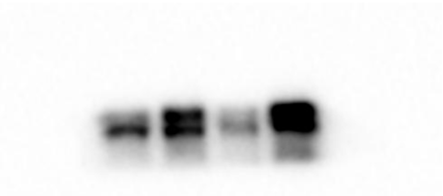

B-catenin

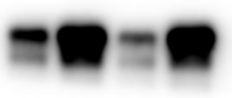

Lamin B1

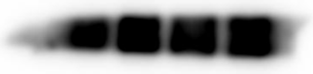

Lamin B1

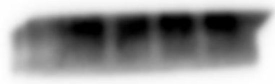

β-actin

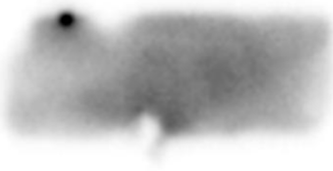

β-actin

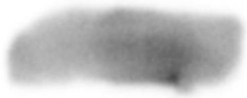

Supplement: Supplementary file 5 — Uncropped western blots [file 41419_2025_7871_MOESM5_ESM.pdf]
